# Fused in sarcoma silences HIV gene transcription and maintains viral latency through suppressing AFF4 gene activation

**DOI:** 10.1186/s12977-019-0478-x

**Published:** 2019-06-25

**Authors:** Simona Krasnopolsky, Lital Marom, Rachel A. Victor, Alona Kuzmina, Jacob C. Schwartz, Koh Fujinaga, Ran Taube

**Affiliations:** 1 The Shraga Segal Department of Microbiology Immunology and Genetics, Faculty of Health Sciences, Ben-Gurion University of the Negev, 84105 Beer Sheva, Israel; 2 Department of Chemistry and Biochemistry, University of Arizona, Tucson, AZ USA; 3 Department of Medicine, University of California, San Francisco, CA USA

**Keywords:** Human immunodeficiency virus, Latency, Transcription, Super elongation complex, AF4/FMR2 family member 4, Positive transcription elongation factor b, Cyclin kinase 9, RNA polymerase II, Elongation factor for RNA polymerase II 2—ELL2, Fused in sarcoma—FUS, Phase separation

## Abstract

**Background:**

The human immunodeficiency virus (HIV) cell reservoir is currently a main obstacle towards complete eradication of the virus. This infected pool is refractory to anti-viral therapy and harbors integrated proviruses that are transcriptionally repressed but replication competent. As transcription silencing is key for establishing the HIV reservoir, significant efforts have been made to understand the mechanism that regulate HIV gene transcription, and the role of the elongation machinery in promoting this step. However, while the role of the super elongation complex (SEC) in enhancing transcription activation of HIV is well established, the function of SEC in modulating viral latency is less defined and its cell partners are yet to be identified.

**Results:**

In this study we identify fused in sarcoma (FUS) as a partner of AFF4 in cells. FUS inhibits the activation of HIV transcription by AFF4 and ELL2, and silences overall HIV gene transcription. Concordantly, depletion of FUS elevates the occupancy of AFF4 and Cdk9 on the viral promoter and activates HIV gene transcription. Live cell imaging demonstrates that FUS co-localizes with AFF4 within nuclear punctuated condensates, which are disrupted upon treating cells with aliphatic alcohol. In HIV infected cells, knockout of FUS delays the gradual entry of HIV into latency, and similarly promotes viral activation in a T cell latency model that is treated with JQ1. Finally, effects of FUS on HIV gene transcription are also exhibited genome wide, where FUS mainly occupies gene promoters at transcription starting sites, while its knockdown leads to an increase in AFF4 and Cdk9 occupancy on gene promoters of FUS affected genes.

**Conclusions:**

Towards eliminating the HIV infected reservoir, understanding the mechanisms by which the virus persists in the face of therapy is important. Our observations show that FUS regulates both HIV and global gene transcription and modulates viral latency, thus can potentially serve as a target for future therapy that sets to reactivate HIV from its latent state.

**Electronic supplementary material:**

The online version of this article (10.1186/s12977-019-0478-x) contains supplementary material, which is available to authorized users.

## Background

Early studies on the regulation of gene transcription of the proviral human immunodeficiency virus (HIV) have laid the foundations to our current understanding of how metazoan transcription elongation is regulated [1]. Following the recruitment of RNA Polymerase II (RNAPII) to the viral promoter and initiation of transcription, RNAPII associates with pause-inducing factors, DRB sensitivity inducing factor (DSIF) and negative elongation factors (NELF), and pauses at 25–50 nucleotides downstream of the mRNA transcription starting site (TSSs) [2–4]. Paused RNAPII remains stable with the nascent RNA, but can fully resume productive transcription elongation upon recruitment of the super elongation complex (SEC) [1, 5–17]. For this step to be efficiently executed, the viral Trans-Activator of Transcription, Tat, acts as a master regulator of transcription elongation by tethering SEC to the HIV trans-activator response (TAR) stem-loop RNA on the viral short transcripts, and synergistically enhancing RNAPII pause-release and elongation. Within SEC two elongation factors, positive transcription elongation factor b (P-TEFb) and elongation factor for RNA polymerase II 2 (ELL2) are key components. In P-TEFb, the Cdk9 kinase phosphorylates NELF and DSIF and antagonizes their inhibitory effects. Cdk9 also phosphorylates the serine 2 of the heptapeptide repeats (YSPTSPS) of the C-terminal domain (CTD) of RNAPII to enhance elongation of transcription. Simultaneously, ELL2 stimulates the processivity of RNAPII through suppressing its transient pausing [1, 2, 18–22]. While HIV recruits SEC via Tat, cells use other mechanisms to bring the elongation machinery to their promoter. The YEATS domain of ENL/AF9 brings SEC to chromatin via the human polymerase-associated factor complex (PAFc) [23, 24], while Brd4 recruits P-TEFb to acetylated chromatin, competing with Tat and inhibiting HIV transcription [25, 26]. SEC also recruits P-TEFb to genes via interactions with Med26 of the mediator [27, 28]. In SEC, the AFF1-4 proteins of the AF4/FMR2 family each act as a scaffold that bridges the complex to P-TEFb, forming a bi-functional complex that synergistically triggers transcription elongation by Pol II [29–33]. AFF proteins integrate within SEC as homo-dimers, or heterodimers, forming alternative complexes that also include a minor complex that potentially modulates HIV latency [34]. In recent years the implementation of high-resolution genome-wide studies further strengthen the current model for RNAPII pausing, release and elongation, reinforcing what was already established for HIV [1, 6, 16, 35–37]. Nevertheless, despite significant progress in understanding the molecular mechanisms that drive RNA pause-release and elongation of transcription, the mechanisms that control SEC functions are less defined and the search for its novel factors that can modulate its function is yet to be completed.

Besides being a model for studying eukaryotic transcription control, there is a clinical significance in studying transcription control of HIV. This step of the virus life cycle is a crucial event in establishing the latent HIV reservoir that harbors transcriptionally repressed virus and is primarily resides in resting CD4+ cells which are resistant to therapy [38, 39]. Thus, despite the introduction of antiretroviral therapy (ART), complete viral eradication remains out of reach [40–45]. While development of novel therapeutic strategies to eliminate the latent viral reservoir is a widely recognized goal, the knowledge on the molecular events that establish and maintain this state is limited. Extensive efforts are being made in optimizing new approaches that will activate the virus without affecting global cell activation and allow subsequent kill of infected cells by standard therapy [44, 46, 47].

Like other proteins of the FET family (FUS, EWSR1 and TAF15; Ewing’s sarcoma (EWS) and TAF15 (TATA-binding protein-associated factor), mutations in fused in sarcoma (FUS) are directly linked with protein aggregation in amyotrophic lateral sclerosis (ALS) and frontotemporal dementia patients. FUS, was first identified in human myxoid and round cell liposarcomas as an oncogenic fusion protein with a stress-induced DNA-binding transcription factor, CCAAT enhancer-binding homologous protein (CHOP, also known as GADD153 or DDIT3) [48, 49]. FUS binds RNA and consists of low complexity (LC) motifs that are important for its functions and for its ability to accumulate into phase separation structures [50–52]. FUS is also involved in regulating gene expression, coupling transcription to splicing via mediating interactions between RNAPII and U1 snRNP [53–56]. However, while FUS interactome has been extensively defined and overlaps with that of RNAPII and the transcription machinery, the functional significance of these interactions has yet to be established and a role for HIV transcription has not yet defined [57]. Knockdown (KD) of FUS promotes a small increase in RNAPII traveling ratios at FUS-bound genes [54]. In addition, ChIP-seq and CLIP-seq analysis of RNAPII in FUS KD neuronal cells demonstrated that FUS is clustered around alternative polyadenylation (APA) sites of nascent RNA. The relative positioning of FUS and APA sites determines the length of the mRNA and the interactions of FUS with CPSF160 [58]. Finally, loss of FUS also leads to accumulation of a phosphorylated Ser2 of the CTD of RNAPII near TSSs of genes that are enriched with FUS. Indeed, in the presence of FUS, the kinase activity of P-TEFb and Cdk12 toward the CTD is specifically inhibited [54].

In this study we employed immuno-purification (IP) followed by mass-spectrometry (MS) to pull-down cell partners of AFF4. Our work identified FUS as a binding partner of AFF4. FUS silenced transcriptional activation from the HIV promoter both in the presence or absence of Tat expression, and restricted AFF4 and Cdk9 occupancy on the viral promoter. Depletion of FUS expression exhibited reversal effects, and led to stimulation of HIV gene transcription. Importantly, knockout of FUS delayed the progressive entry of HIV into a latency state in infected T cells. In another T cell latency model, depletion of FUS expression also enhanced the activation effects of the BET bromodomain inhibitor JQ1. In addition, live imaging analysis demonstrated that FUS co-localized with AFF4 in the cell nucleus, exhibiting a punctuated expression pattern. Upon treating cells with Hexanediol, which disrupts phase separation structures, nuclear AFF4-FUS co-localization was disrupted, and proteins migrated to the cytoplasm. Finally, effects of FUS in modulating the transcription from the HIV promoter were also exhibited genome-wide, as elevated occupancy levels of AFF4 and Cdk9 were detected around gene promoters that were upregulated following knockdown of FUS expression. Overall, we conclude that FUS silences HIV transcription and modulates viral latency through its recruitment to the host elongation machinery and restricting SEC/P-TEFb on the viral promoter.

## Results

### Identification of AFF4-associating proteins in cells

**Fig. 1:**
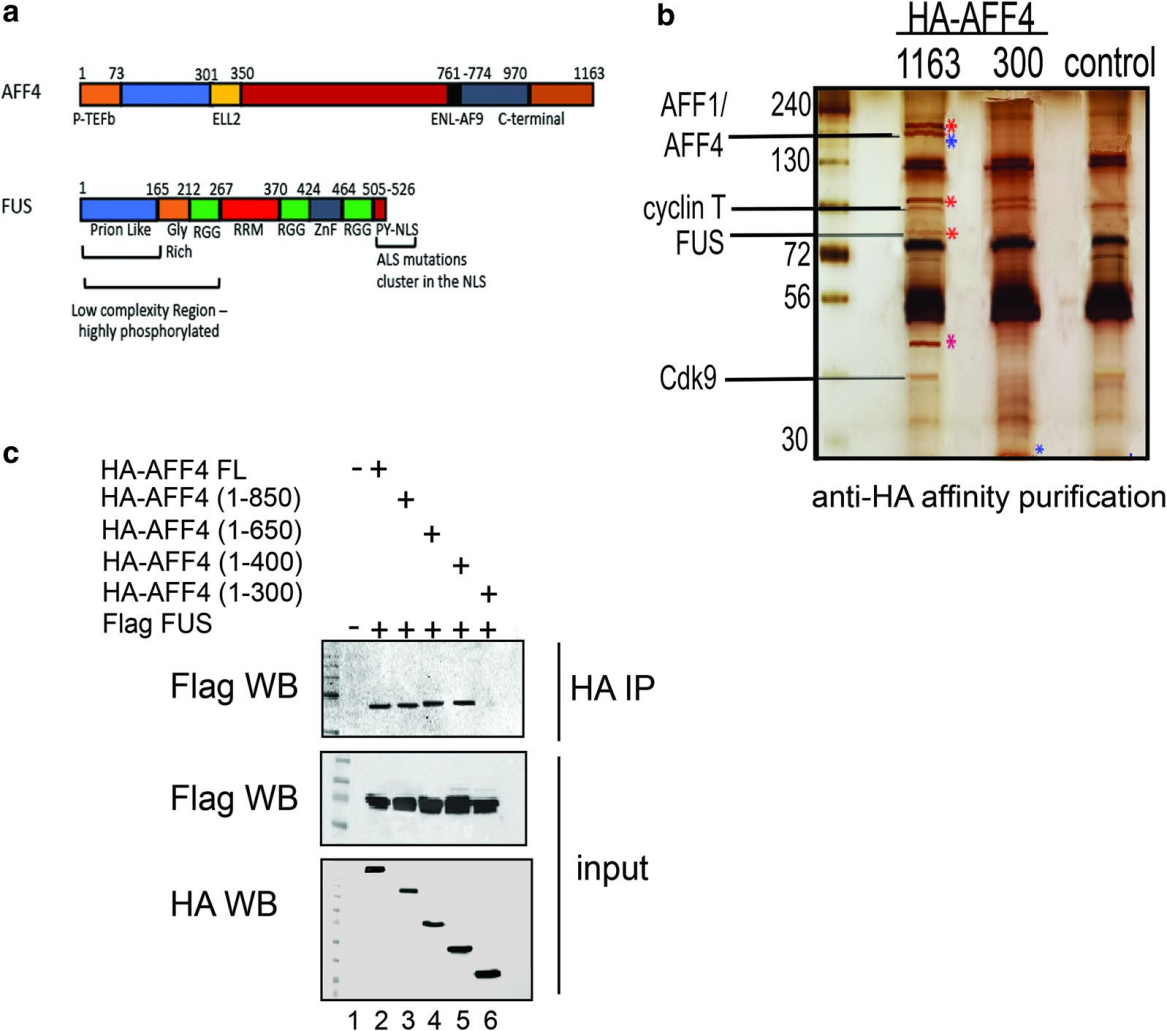
Characterization of AFF4-associated proteins. **a** Schematic domain organization of human AFF4 and FUS proteins. AFF4 serves as a scaffold for the assembly of SEC subunits including the elongation transcription complexes ELL1/2 and P-TEFb [83]. At its N-terminal region it consists the P-TEFb binding domain (orange); the ELL domain (yellow); ENL/AF9 (black); and a C-terminal motif (orange). In FUS, the N-terminal of the protein exhibits low sequence complexity (blue). The RGG domains contain a triplet repeat motif of arginine–glycine–glycine (green). FUS also contains an RNA-recognition motif (RRM) (red), a zinc-finger domain (ZnF) gray), and a proline–tyrosine nuclear localization signal (PY–NLS; dark red) [55]. **b** Purification of AFF4 protein partners by affinity purification. Control HEK293T cells, or cells expressing either Full length HA-AFF4 (1–1163) or its truncated form HA-AFF4 (1–300) were subjected to HA-epitope-tagged immuno-purification (IP) with anti-HA antibody. IP samples were separated by SDS-PAGE and visualized by silver staining. Cellular partners of HA-AFF4 proteins were also identified by tandem mass spectrometry. Previously confirmed SEC/P-TEFb partners as well as newly identified partners like FUS were recovered by our MS analysis (also see Additional file 1: Figure S1, Additional file 2: Table S1 and Additional file 3: Table S2 for quantitative analysis of the MS results). Red asterisks indicate potential novel AFF4 partners, which FUS is one of them: Blue asterisks point to the full-length and truncated AFF4 (1–300) proteins. **c** N-terminus region of AFF4 associates with FUS in cells. Western blot analysis of immuno-precipitation (IP) samples defining the regions of AFF4 that mediate association with the FUS in cells. Lysates from HEK293T cells expressing Flag-FUS and the indicated HA-AFF4 proteins were IP with anti-HA IgG. IP. IP and input (5%) samples were analyzed by SDS-PAGE followed by western blot analysis with anti-HA or anti Flag antibodies

To isolate cellular partners of AFF4 that potentially play a role in regulating the functions of SEC in gene transcription, we took a proteomic approach and expressed full length HA-AFF4 (1–1163), or its truncated form HA-AFF4 (1–300) in HEK293T. We further conducted immuno-affinity purification (IP) of cell lysate using anti-HA antibody, and IP samples were resolved on SDS-PAGE and visualized by silver-staining (Fig. 1b). In addition, samples were subjected to mass spectrometric (MS) analysis to identify the recovered proteins that co-purified with AFF4 [see Additional file 1: Figure S1—quantitative MS analysis; Additional file 2: Table S1 (MS_AFF4-full length-FL) and Additional file 3: Table S2 (MS_AFF4-300)]. Our analysis identified previously known partners of SEC such as Cdk9 and cyclin T1 that associated with full-length AFF4 and AFF4-(1–300), but not with the control cells that did not express HA-AFF4 proteins, validating our purification scheme (Fig. 1b; Additional file 1: Figure S1). Interestingly, among the top hits that were recovered with full-length HA-AFF4, but not HA-AFF4-(1–300) or control cells, was an approximately 70 kDa protein that was identified by the MS analysis as Fused in Sarcoma, FUS (Fig. 1b; Additional file 1: Figure S1). FUS is a member of the FET protein family, and associates with RNAPII-CTD in an RNA-dependent manner [48, 54, 55, 59–61]. FUS has also been recently reported to localize within nuclear phase separated assemblies, while mutations in its N-terminal low complexity (LC) regions (Fig. 1a) impair this distribution, linking FUS to the establishment of neurodegenerative disease like ALS [50, 52, 62–64].

We also aimed to confirm that FUS associates with AFF4 in cells, and to define the regions of AFF4 that interact with FUS. Flag-FUS and full length HA-AFF4 or its truncated proteins including HA-AFF4 (1–850) (1–650), (1–400), (1–300) were over-expressed in cells, and cell lysates were subjected to IP with HA-antibody, followed by western blotting with anti-Flag antibody (Fig. 1c). Our analysis demonstrated that while HA-AFF4 full-length (1–1163) and truncated proteins HA-AFF4 (1–850) (1–650) and HA-AFF4 (1–400) associated with Flag-FUS, a shorter form of HA-AFF4 (1–300) could not associate with Flag-FUS (Fig. 1c). We conclude that N-terminal residues of AFF4 between positions 300–400 are required for association with FUS.

### FUS inhibits HIV transcription from the viral promoter

**Fig. 2:**
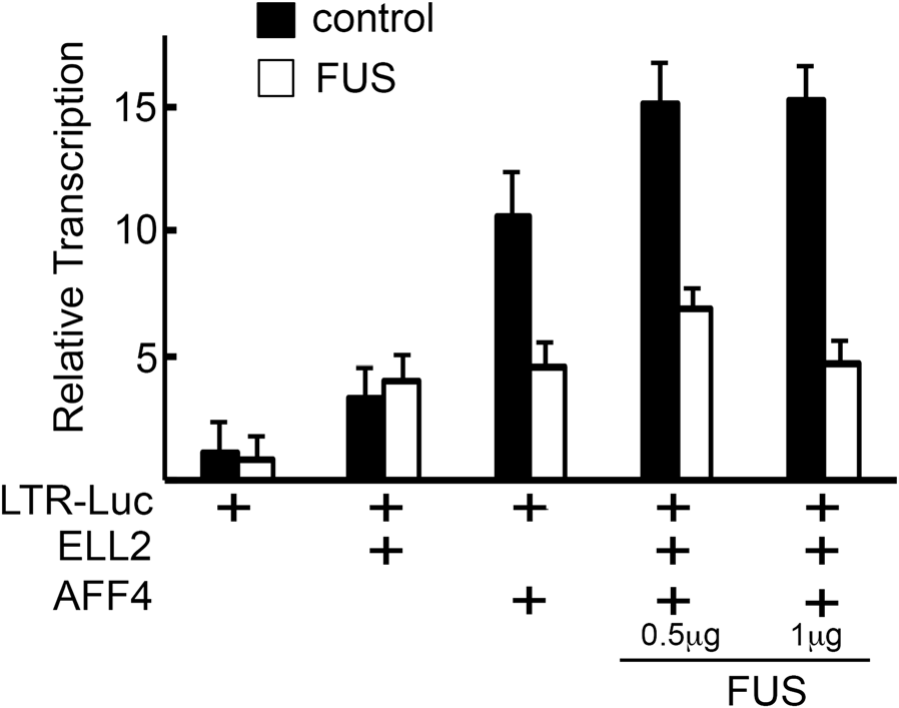
FUS inhibits activation from the HIV promoter by AFF4 and ELL. Cells expressing AFF4, ELL2 or both proteins were monitored for their ability to activate the HIV promoter in the presence or absence of FUS. Indicated concentrations of FUS expression plasmid were also used. Relative transcription corresponds to luciferase readings relatively to control cells that express the LTR-Luc - set to 1. Readings are representative of three independent experiments. The error bars represent mean ± SD from three independent reactions

Much of our current understanding of the functions of P-TEFb and SEC comes from pivotal work on the control of transcription elongation of HIV, which is heavily depended on the activity of these two transcription elongation complexes [11, 38]. To elucidate the role of FUS in HIV transcription, we initially examined its effects on AFF4 and ELL2-mediated activation of HIV transcription in the presence or absence of FUS. Previous results reported that AFF4 activates transcription from the HIV promoter and cooperates with ELL2 to synergies basal but not Tat-dependent HIV transcription [22]. Thus, the LTR-Luciferase (Luc) reporter cassette was expressed in HEK cells with AFF4 or ELL2, monitoring their effects on HIV LTR gene transcription (Fig. 2). Our results confirmed that ELL2 activated HIV transcription from the LTR promoter fourfold, while AFF4 was more potent and stimulated LTR-Luc transcription 10 fold. Co-expression of both ELL2 and AFF4, further enhanced HIV gene transcription up to 15 fold relative to basal activation. Significantly, expression of FUS, inhibited AFF4-mediated activation of HIV gene transcription twofold. FUS also exhibited silencing effects on HIV transcription in the presence of both AFF4 and ELL2 (Fig. 2).

**Fig. 3:**
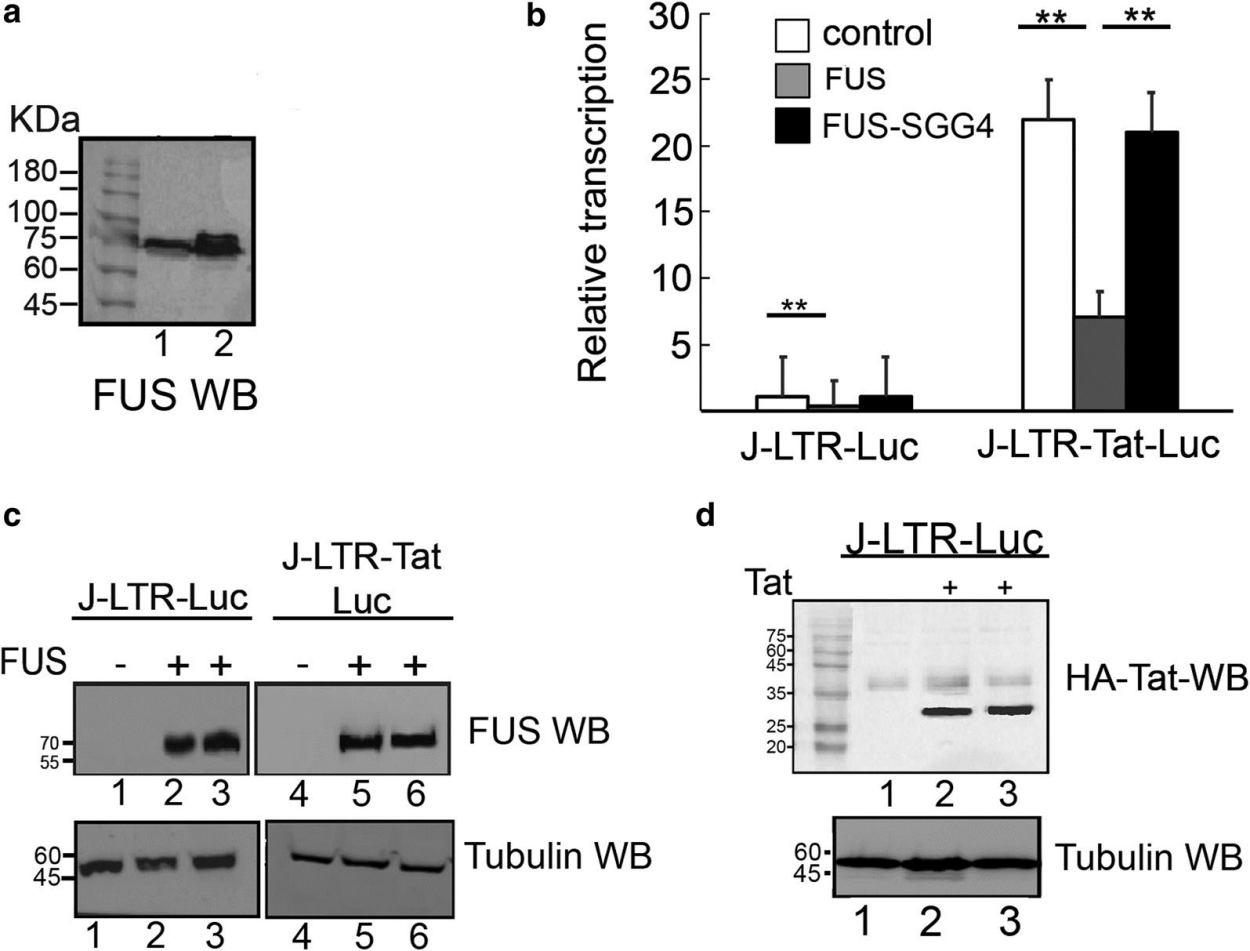
FUS silences gene transcription from the HIV promoter. **a** FUS expression in Jurkat cells. Western Blotting analysis confirming endogenous expression of FUS in J-LTR-Luc cells, (lane 1) and expression of Flag-FUS in J-LTR-Luc-FUS cells (lane 2) using FUS IgG. **b** FUS silences transcription from the HIV promoter. Jurkat (J)-LTR-Luc and J-LTR-Tat-Luc cells that stably express Tat, were monitored for their LTR luciferase readings in the absence or presence of FUS expression (gray bars), or its SGG4 mutant that does not bind RNA (black bars). Relative transcription corresponds to luciferase readings relatively to control Jurkat cells that express the LTR-Luc reporter gene - J-LTR-Luc - set to 1 (white bars). Readings are representative of three independent experiments. The error bars represent mean ± SD from three independent reactions. Asterisks indicate levels of statistical significance as calculated by two-tailed student T test (**p ≤ 0.01). **c** Western Blot analysis confirming Flag-FUS (lanes 2**–**3) and SGG4 (5**–**6) expression in J-LTR-Luc and J-LTR-Tat-Luc cells using anti-Flag IgG. Lanes 1 and 3 represent cells that do not express Flag-FUS. Lower panel represent tubulin western blot for monitoring loading control. **d** Western Blot analysis to confirm equal expression of Tat in J-LTR Tat-Luc and J-LTR-Tat-Luc-FUS (lanes 2 and 3) and compared to J-LTR -Luc that do not express Tat (lane 1). Lower panel represent tubulin western blot for loading control

We next aimed to monitor the effects of FUS on overall HIV gene transcription in Jurkat T cells, which are a more natural cell target of HIV. Western Blot analysis confirmed FUS expression in these cells (Fig. 3a, lane 1; lane 2 monitored Flag FUS expression). We then analyzed the role of FUS in regulating HIV gene transcription in Jurkat (J)-LTR-luciferase T cells (J-LTR-Luc) that harbor an integrated luciferase reporter gene under the control of the HIV promoter (Fig. 3b). J-LTR-Luc cells were transduced with lentivirus that drive the expression of Flag-FUS and a GFP reporter gene that was translated via an IRES sequence. Transduced cells were further sorted by FACS based on their GFP expression—generating J-LTR-Luc-FUS cells and their Flag-FUS expression was validated by western blotting with anti-Flag antibody (Fig. 3c). In addition, we also generated J-LTR-Luc cells that stably expressed a Flag-FUS mutant that does not bind RNA, as it consists of arginine to serine mutations in RGG1 2 and 3 of FUS (J-LTR-Luc-FUS SGG4) [65]. Analysis of HIV LTR driven luciferase activity in control J-LTR-Luc and in J-LTR-Luc FUS cells demonstrated that ectopic expression of FUS moderately inhibited gene transcription from the HIV promoter (twofold), relative to control J-LTR-Luc cells that did not over-expressed FUS (compare gray bars to white bars in J-LTR-Luc FUS cells relative to control J-LTR-Luc cells; Fig. 3b). Expression of the SGG4 FUS mutant in J-LTR-Luc cells, did not repressed HIV-mediated luciferase activity and transcription levels returned to those displayed by the wild type J-LTR-Luc control cells (black bars *versus* white bars), implying that the RNA binding of FUS is required for the ability of FUS to repress HIV transcription.

FUS effects were also tested in Jurkat (J)**—**LTR-Tat-Luc cells that stably express the Tat protein under the HIV LTR promoter. To generate these cells, J-LTR-Luc were transduced with lentivirus encoding Tat-BFP (LTR-HA-Tat-BFP), following by cell sorting based on BFP expression. J-LTR-Tat-Luc cells were further transduced with lentivirus expressing Flag-FUS (or FUS-SGG4) as described above, to generate J-LTR-Tat-FUS J-LTR-Tat-Luc FUS SGG4. FUS expression was confirmed by western blotting with anti-Flag antibody (Fig. 3c). As expected, Tat expression enhanced gene transcription activity from the HIV promoter to about 22 fold relative to control J-LTR-Luc cells that did not express Tat (compare white bars; Fig. 3b). In contrast, following ectopic expression of FUS, Tat transactivation was repressed about threefold relative to J-LTR-Tat-Luc (compare white bars relative to gray bars of J-LTR-Tat-Luc-FUS; Fig. 3b). No inhibition of HIV transcription was observed upon ectopic expression of the FUS-SGG4 mutant (Fig. 3b; black bar). To verify that the effects of FUS on HIV transcription were direct and not stem from different levels of Tat expression we also monitored HA-Tat expression levels by western blotting, confirming equal HA-Tat expression levels in J-LTR-Tat-Luc and J-LTR-Tat-Luc-FUS cells, (Fig. 3d). We thus conclude that FUS inhibited both basal (Tat independent) and Tat dependent HIV transcription.

### Knockout of FUS expression activates gene transcription from the HIV promoter

**Fig. 4:**
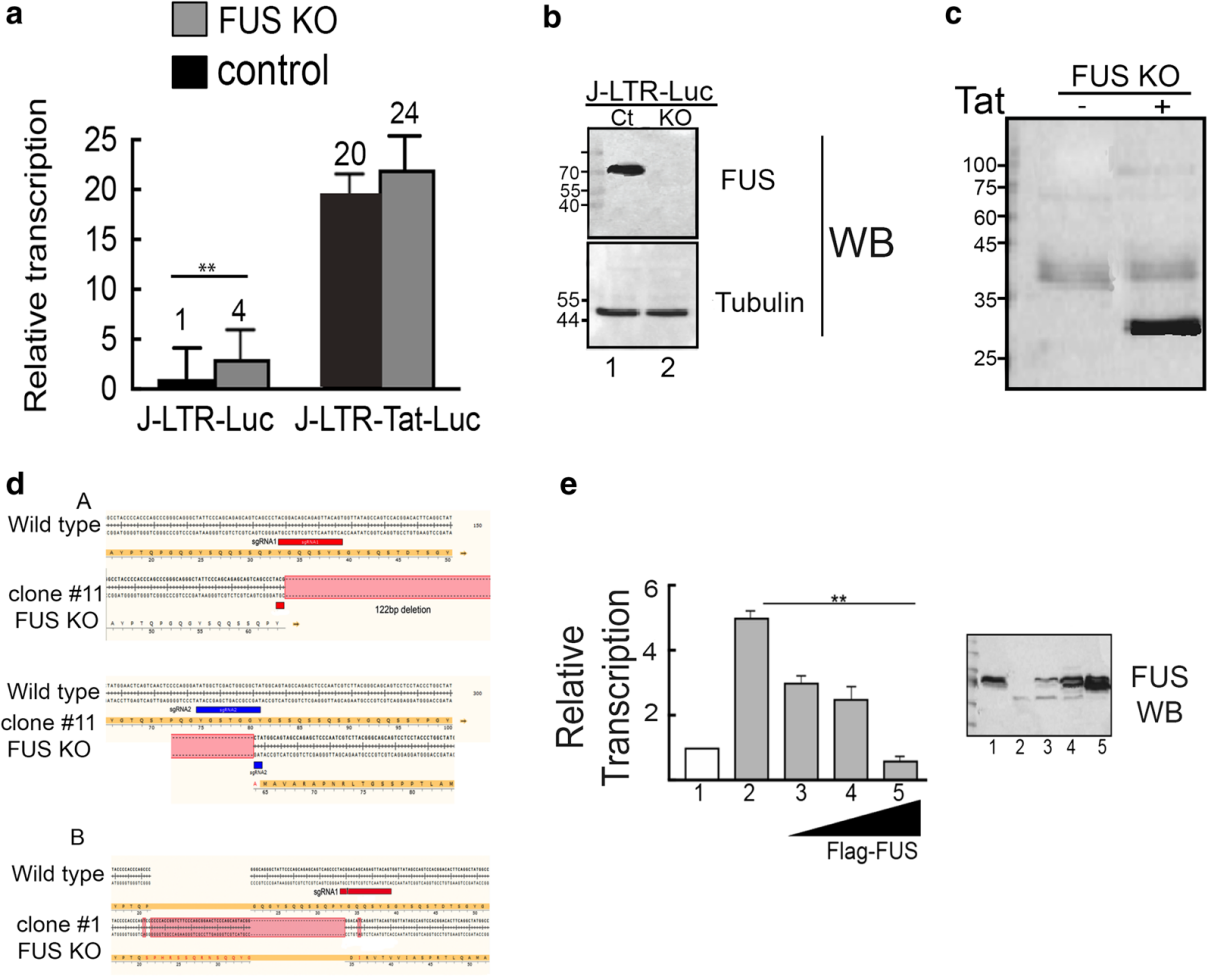
Depletion of FUS expression activates transcription of HIV. **a** Depletion of FUS activates HIV gene activation. Luciferase readings were determined in J-LTR-Luc FUS KO and J-LTR-Tat-FUS KO cells, where FUS expression was depleted. To obtain these cells, Jurkat (J)-LTR-Luc were transduced with lentivirus driving the expression of Cas9/sgRNA (FUS specific) and following puromycin drug selection, mono-clonal cells where FUS expression was knockout (KO) were obtained (J-LTR-Luc-FUS KO; gray bar). Control cells were similarly generated, where their CRISPR/sgRNA vector harbored a scrambled sgRNA (black bar). To generate J-LTR-Tat-FUS KO, a selected J-LTR Luc FUS KO clone was further transduced with lentivirus expressing HA-Tat-BFP, and cells were sorted based on their BFP expression (black bar). Luciferase activity was monitored 48 h post transduction according to the manufacturer protocols. Data are presented relatively to luciferase readings in control cells J-LTR-Luc—set to 1, and are representative of three independent experiments. The error bars represent mean ± SD from three independent reactions. Asterisks indicate levels of statistical significance as calculated by two-tailed student T test (**p ≤ 0.01). When asterisks are not shown, no statistically significant difference was observed. **b** Western Blot analysis of J-LTR-Luc FUS KO cells using FUS antibody, confirming depletion of FUS expression (lane 2). Endogenous expression of FUS in control J-LTR-Luc cells is also presented (ct; lane 1). **c** Western Blot analysis of J-LTR-Tat-Luc FUS KO cells confirming HA-Tat expression. J-LTR-Luc-FUS KO cells were transduced with lentivirus that drive the expression of HA-Tat. Cells were sorted based on their BFP expression (linked to Tat via IRES). Sorted cells were then harvested and subjected to luciferase assay and western blotting using an HA IgG. Tat expression in control J-LTR-Tat Luc cells was also monitored (lane 1). **d** Characterization of J-LTR-Luc-FUS knockout clones. Genotyping of genomic DNA isolated from the two J-LTR-Luc-FUS KO clones, where the gene encoding for FUS was disrupted following the introduction of Cas9/sgRNA. Presented is the nucleotide and amino acid residues of FUS around the region where the sgRNA oligos was located. Two independent clones are presented (**a** and **b**), where deletions were generated around the sgRNA sequence targets. **a**—in Clone #11 two sgRNA were used (sgRNA 1 and 2) and generated a 122 bp gap; **b**—in Clone #1, a single sgRNA was used (sgRNA 1) formed a short deletion as well. **e** Overexpression of FUS restores HIV gene silencing in FUS KO cells. J-LTR-Luc-FUS KO cells were transduced with increasing MOI of lentivirus that drive the expression of Flag-FUS. Cells were sorted based on their GFP (linked to FUS via IRES) intensities to obtain different levels of FUS expression in cells. Sorted cells were further grown, harvested and subjected to luciferase assay according to the manufacturer protocol. Results are presented relatively to luciferase readings in J-LTR-Luc control cells that express scrambled sgRNA—set to 1. Error bars show mean ± SD from three independent reactions. Asterisks indicate levels of statistical significance as calculated by two-tailed student T test (*p* < 0.01). Also presented is a western blot verifying increasing amounts of FUS expression. Control J-LTR-Luc express endogenous FUS (lane 1), while J-LTR-Luc-FUS KO cells do not (lane 2). J-LTR-FUS-KO that express increasing concentrations of Flag FUS are also presented (lanes 3–5)

We next depleted the expression of endogenous FUS in Jurkat (J)-LTR-Luc, using CRISPR/Cas9 (Fig. 4). J-LTR-Luc were transduced with lentivirus that drive the expression of Cas9 and several small guides sgRNA that specifically target FUS. Cells were subjected to puromycin selection, and knockout (KO) of FUS expression was verified by western blotting, as well as by genomic sequencing (Fig. 4b, shows a representative WB of a J-LTR-Luc-FUS KO clone; sequence analysis near the sgRNA position site of two representative clones are also shown in Fig. 4d). Control J-LTR-Luc cells that encoded Cas9 and a control scrambled sgRNA were also generated. As shown in Fig. 4, upon FUS KO, transcription from the LTR HIV promoter in J-LTR-Luc FUS KO was upregulated fourfold relative to control J-LTR-Luc cells that expressed scrambled sgRNA (compare the black bar of control cells to the gray bar of J-LTR-Luc-FUS KO cells—Fig. 4a). Effects of FUS KO on gene transcription from the HIV LTR promoter were also tested in the presence of Tat. Herein, J-LTR-Luc FUS KO were further transduced with lentivirus expressing HA-Tat BFP to generate J-LTR-Tat-FUS KO cells. Tat expression was confirmed by western blotting (Fig. 4c). As expected, upon Tat expression, HIV gene transcription in J-LTR-Tat Luc cells was stimulated up to 20 fold relative to control J-LTR-Luc cells (compare J-LTR-Luc and J-LTR-Tat-Luc black bars; Fig. 4a). Upon FUS KO, Tat transactivation was also increased, reaching similar levels as in the control cells that expressed the scramble sgRNA. J-LTR-Tat-Luc FUS KO exhibited a 24 fold increase in HIV transcription relative to J-LTR-Luc control cells (gray bar in J-LTR-Tat-Luc-FUS KO, relative to black bar in J-LTR-Luc). We thus conclude that despite loss of FUS, Tat transactivation still reached optimal levels.

To further strengthen the inhibitory effects of FUS on HIV transcription, we examined whether the silencing of HIV gene transcription by FUS in J-LTR-Luc-FUS KO cells could be reversed by re-introducing exogenous FUS. J-LTR-Luc FUS KO cells were thus transduced with increasing MOI of lentivirus that drive the expression of Flag-FUS-IRES-GFP. Cells were sorted based on their GFP expression by FACS and were grouped based on their GFP intensity. Sorted cells were then harvested and subjected to luciferase assay, monitoring effects on HIV gene transcription (Fig. 4e). As seen above, FUS KO activated HIV transcription—fivefold (Fig. 4e, lane 2). Increasing expression levels of FUS in J-LTR-FUS KO cells led to inhibition of viral transcription (Fig. 4e, lanes 3–5).

### FUS associates with the HIV promoter through TAR and restricts the occupancy of AFF4 and Cdk9 on the HIV promoter

**Fig. 5:**
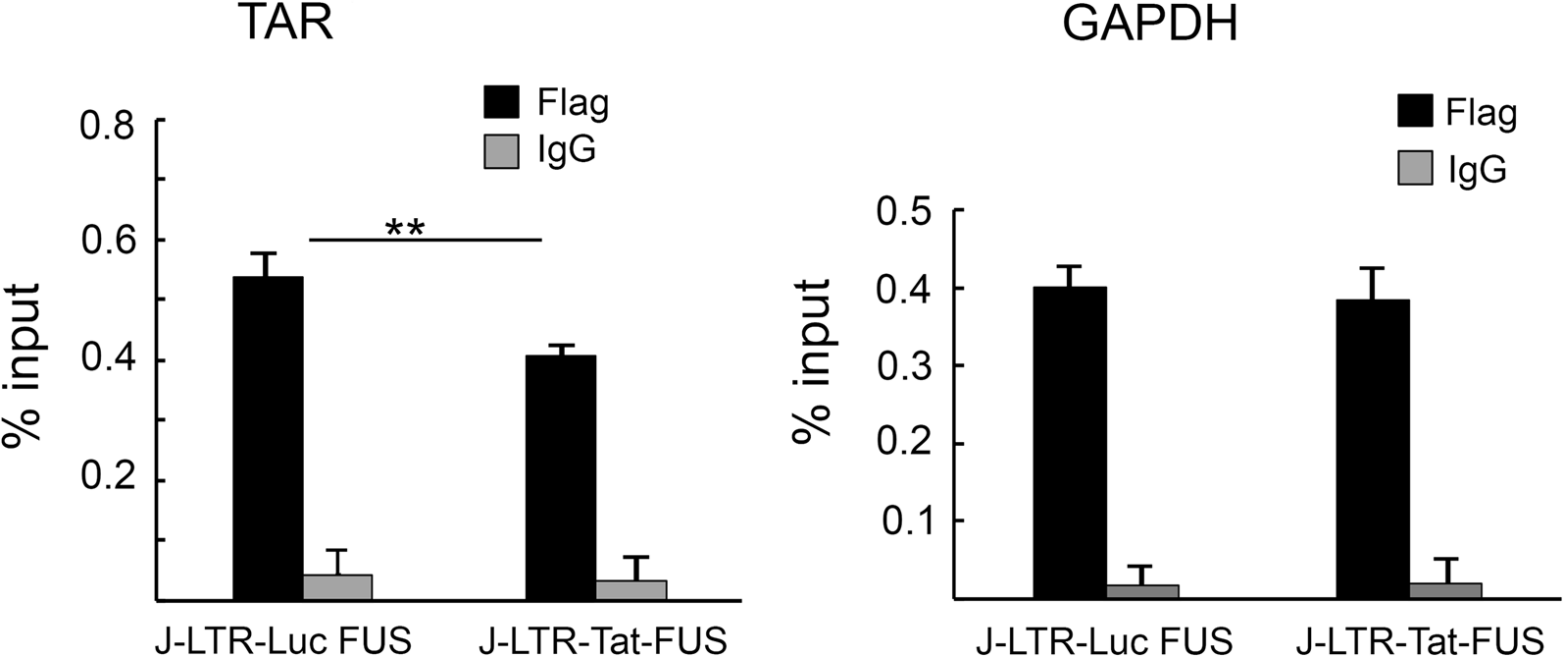
FUS non-specifically associates with TAR RNA. Jurkat (J)-LTR-Luc-FUS and J-LTR-Tat-FUS cells that harbor the integrated HIV-LTR-Luc reporter and also over-express Flag-FUS were subjected to RNA immunoprecipitation (RIP) qPCR. Cell lysate was immune-precipitated with either anti-Flag-IgG (black bars), or control non-specific IgG (gray bars). RNA was then extracted from IP or input (10%) samples with Trizol, followed by reverse transcription and amplification with primers that target the indicated RNA. qPCR reactions on samples were performed in triplicates and presented as percentage from input ChIP material. The error bars represent mean ± SD of the triplicate independent qPCR reactions. Asterisks indicate levels of statistical significance calculated by two-tailed student T test (*p* < 0.01)

Through its basic residues RGG/RRM motifs, FUS binds RNA and plays a role in association with the CTD of RNAPII to modulate gene transcription [54, 65]. However, FUS binding to RNA is non-specific as previous reports determine that it exhibits a wide-range affinity to its RNA substrates and possess a general nucleic acid binding activity binding [60, 65]. We thus tested the association of FUS to HIV TAR using RNA-immunoprecipitation (RIP)-qPCR analysis in J-LTR-Luc-FUS cells that stably over-express Flag-FUS (Fig. 5). Cell lysates were immuno-precipitated with anti-Flag or control antibodies, and RNA that was co-immuno-precipitated material was extracted, reverse transcribed and amplified by qPCR using specific primers for TAR. GAPDH RNA was similarly quantified as control (Fig. 5). Our analysis demonstrated that FUS associated with TAR in HIV transduced cells. However, these interactions were not specific, as FUS also associated with GAPDH RNA to similar levels. RIP qPCR experiments were also performed in J-LTR-Tat Luc FUS. Upon Tat expression, FUS association with TAR was slightly, but statistically significant, inhibited, while such inhibition was not displayed when FUS-GAPDH interactions were monitored (Fig. 5). Moreover, our RIP analysis with a FUS mutant that does not bind RNA (FUS SGG4) demonstrated that its association with the TAR HIV promoter were close to background levels (Additional file 1: Figure 2SA). Finally, a Tat mutant that does not bind TAR (Tat 1–48) demonstrated that it cannot compete with FUS on association with TAR RNA (Additional file 1: Figure S2B).

**Fig. 6:**
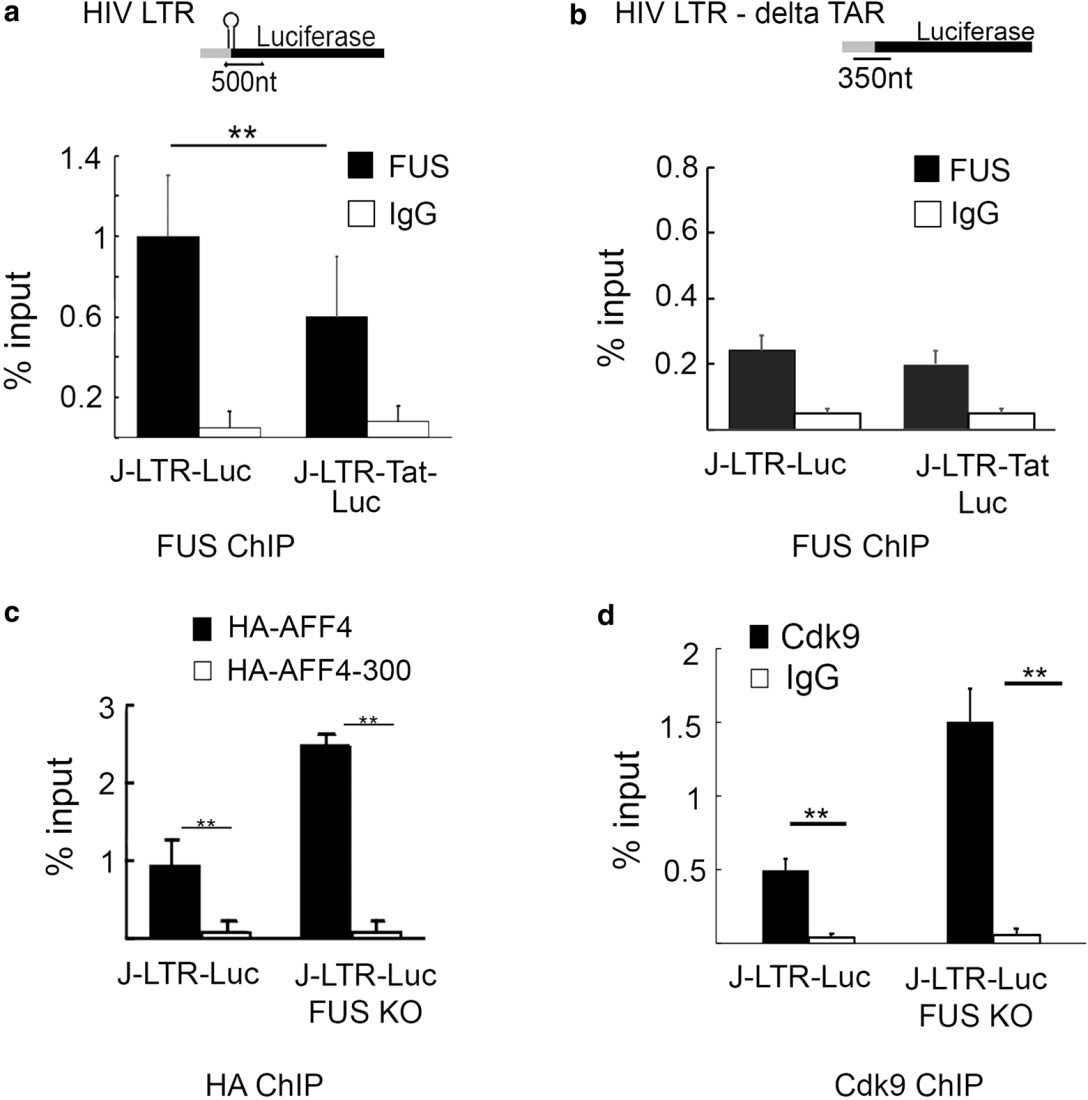
FUS occupies the HIV promoter and its depletion enhances the recruitment of SEC and P-TEFb to the viral promoter. **a** + **b** FUS occupancies the HIV promoter. ChIP material was isolated from Jurkat (J)-LTR-Luc or J-LTR-Tat-Luc cells, where the LTR promoter either harbors TAR (**a**), or consists of a LTR-delta TAR (**b**). Cells were subjected to FUS-IP using FUS antibody. qPCR on IP samples was conducted with a pair of primers located on the HIV promoter and signals were presented as percentage from input. The error bars represent mean ± SD from three independent qPCR reactions. Asterisks indicate levels of statistical significance calculated by two-tailed student T test (*p* < 0.01). **c** FUS restricts AFF4 occupancy on the HIV promoter. ChIP material from Jurkat (J)-LTR-Luc cells and J-LTR-Luc-FUS KO, where FUS expression was depleted and also express HA-AFF4 or HA-AFF4-300, was subjected to immune-precipitated with anti-HA-antibody or control antibody followed by qPCR with primers located on the HIV promoter. Data are presented as percentages of the input DNA, and representatives of three independent experiments. The error bars represent mean ± SD from three independent qPCR reactions. Asterisks indicate levels of statistical significance as calculated by two-tailed student T test (**p ≤ 0.01). **d** FUS modulates Cdk9 occupancy on the HIV promoter. ChIP-qPCR analysis of J-LTR-Luc, or J-LTR-Luc-FUS-KO cells were performed by using anti-Cdk9 IgG. As control, IP on ChIP material was also performed with non-specific IgG. qPCR analysis was performed with the primers located on the HIV promoter (**a**). Signals present percentage from input DNA, and results are representative of three independent experiments. The error bars represent mean ± SD from three independent qPCR reactions. Asterisks indicate different levels of statistical significance as calculated by two-tailed student T test (**p ≤ 0.01)

As our results suggested that FUS associates with AFF4 and also interacts with TAR, we next monitored FUS occupancy on the HIV promoter by employing quantitative chromatin IP (ChIP) - qPCR - in J-LTR-Luc or J-LTR-Tat Luc cells (Fig. 6). ChIP material was isolated from these cells and subjected to IP with anti-FUS or control anti-rabbit antibodies, and qPCR analysis was performed using primers located on the HIV promoter near its TSS (Fig. 6). Our analysis suggested that FUS was specifically recruited to the HIV promoter, as a control IgG showed a very low ChIP qPCR signal. Moreover, the role of TAR on FUS occupancy was also determined by comparing FUS occupancy on an integrated viral promoter that is deleted with TAR (delta TAR). Our results showed that TAR deletion lowered the levels of FUS occupancy on the HIV promoter relatively to FUS levels when (Fig. 6a + b). Moreover, FUS occupancy on an HIV-LTR TAR promoter was reduced in J-LTR-Tat FUS cells that express Tat, implying of the ability of Tat to compete with FUS on its occupancy on the HIV promoter as it potentially masks the RNA target (Fig. 6a). No effects of Tat on FUS occupancy were detected when a delta-TAR promoter was used in the qPCR assay as Tat cannot bind the HIV promoter without TAR (Fig. 6b). We further examined by ChIP-qPCR the effects of FUS in recruiting AFF4 and P-TEFb to the HIV promoter. Experiments were performed in J-LTR-FUS KO cells that transiently expressed either HA-AFF4-FL (full length), or HA-AFF4 (1–300) using anti-HA or control antibodies (Fig. 6c). Our results showed that KO of FUS expression elevated the occupancy of full-length AFF4 on the viral promoter compared to control cells that expressed endogenous FUS. In addition, regardless of FUS expression, HA-AFF4-(1–300) could not occupy the HIV promoter (Fig. 6c). The occupancy of Cdk9 in Jurkat (J) -LTR-Luc, or J-LTR-Luc-FUS KO was also monitored by ChIP-qPCR using anti-Cdk9 or control antibodies (Fig. 6d). Our analysis demonstrated that in J-LTR-Luc-FUS KO cells, upon FUS KO, Cdk9 occupancy on the HIV promoter was elevated, relative to that in control J-LTR-Luc cells. We conclude that FUS modulates SEC/P-TEFb occupancy on the HIV promoter, and its KO leads to an increase of SEC/P-TEFb occupancy, resulting in the activation of viral transcription.

### Depletion of FUS expression enhances the reactivation of HIV latency by JQ1

**Fig. 7:**
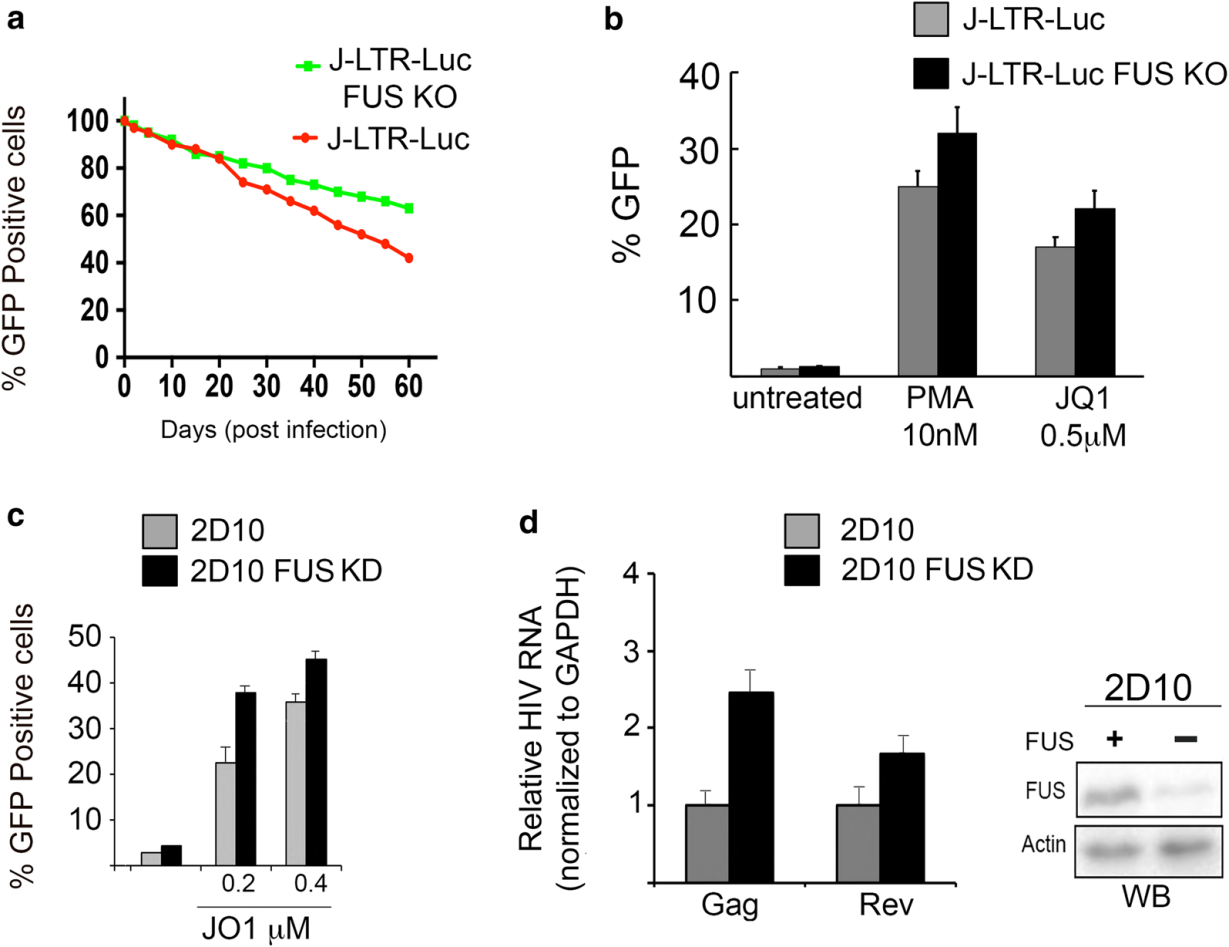
Knockdown of FUS expression enhances reactivation of HIV latency by JQ1. **a** Depletion of FUS delays the establishment of HIV latency. J-LTR Luc (red line) or J-LTR-Luc FUS KO cells (green line) were transduced with pseudotyped HIV-pNL4-GFP virus, where a GFP reporter in inserted instead of the *env* gene. Cells were sorted based on their GFP expression (day 0 post infection) and further grown for the indicated time days post infection to allow them to gradually enter viral latency. GFP expression was monitored at the indicated time points by FACS analysis as a reference for entry into viral latency. **b** Reactivation of latent cells. At 60 d.p.i., transduced J-LTR Luc or J-LTR-Luc FUS KO cells were sorted based on their GFP expression for GFP(−) cells. Cells were then treated for 24 h. with either PMA or JQ1 activators, at the indicated concentrations, and subjected to FACS analysis to monitor their GFP expression, which corresponds to viral reactivation. Error bars indicate mean ± SD from triplicates. **c** Knockdown of FUS expression enhances reactivation of HIV latency by JQ1. 2D10 latent cells were introduced with either FUS-specific or scrambled (control) siRNA oligos. 72-h post transfection, cells were treated with JQ1 in the indicated concentrations. 24 h post treatment HIV gene expression was analyzed by FACS, monitoring d2GFP. **d** HIV RNA levels are elevated upon FUS KD in 2D10 cells and treatment with JQ1. Control 2D10 latent cells (harboring scrambled siRNA), or cells where FUS expression was depleted by specific siRNA were treated with 0.2 μM JQ1 and subjected to RT-qPCR with specific primers amplifying the indicated HIV gene targets - *Gag* and *Rev*. Results are presented relative to GAPDH mRNA levels. Also presented is a western blot confirming KD of FUS expression

Our results indicate that FUS limits the occupancy of AFF4 and P-TEFb on the HIV promoter (Fig. 6) and inhibits viral gene transcription (Figs. 2, 3). As repression of HIV transcription is key for the establishment and maintenance of the HIV latent reservoir, we examined if depletion of FUS expression affects HIV latency. For this J-LTR-Luc cells that harbor an integrated LTR-Luc reporter, or J-LTR-Luc FUS KO cells were transduced with a pseudotyped HIV encoding virus (pHR-GFP). At day 2 post infection, control or FUS KO cells were sorted based on their GFP expression, and GFP(+) cells were further cultured to allow them to progressively enter latency, while monitoring their GFP expression at the indicated time post transduction (Fig. 7a). At 60 d.p.i. (days post infection) both control J-LTR-Luc and J-LTR-Luc-FUS-KO cells entered latency, as detected by the gradual decrease in the GFP expression (Fig. 7a). Significantly, J-LTR-Luc-FUS KO cells exhibited a delay in their entry into viral latency, compared with control J-LTR-Luc cells. Differences in GFP expression between the two cell types were visible as early as 20 d.p.i and at 60 dpi, 80% of LTR-Luc-FUS KO cells expressed HIV-GFP, while only 40% of control cells expressed GFP (Fig. 7a). To ensure that indeed the cells entered latency, pooled GFP(−) cells from either J-LTR Luc or J-LTR-Luc FUS KO were re-sorted at day 60, and the isolated GFP(−) cells were treated with either Phorbol 12-myristate 13-acetate - a PKC agonist (PMA), or JQ1 - a BET bromodomain inhibitor and analyzed by FACS for GFP expression (Fig. 7b). Our results verified that at 60 d.p.i. cells did not express the LTR-GFP. However, following treatment with HIV activators, PMA or JQ1, expression of the integrated LTR-GFP was elevated, implying the activation of latent HIV in these cells (for J-LTR-Luc cells - up to 25% with PMA, and 17% with JQ1; for J-LTR-Luc FUS KO cells 32% with PMA and 22% with JQ1). Overall, we conclude that KO of FUS expression in J-LTR Luc that harbor integrated HIV delays viral entry into a latency state.

We further examined the effects of FUS on reactivation of HIV in a different latency cell model - 2D10. These cells express an attenuated form of Tat (H13L) that activates in cis the expression of an integrated d2GFP reporter inserted in place of the *nef* gene and is regulated under the control of the viral LTR promoter [66]. 2D10 were transfected with either a scrambled, or FUS-specific siRNA and knockdown (KD) of FUS was verified by western blotting. Cells were also analyzed for their HIV gene expression by FACS (Fig. 7c). Our results showed that in 2D10 cells where FUS expression was KD, there was a slight increase in expression levels of HIV-d2GFP, relative to control 2D10 cells that expressed scrambled siRNA, indicating a possible activation of latent cells (Fig. 7c). We also monitored the effects of FUS KD on latent HIV, following reactivation of cells with JQ1—a Bromodomain domain (BET) inhibitor with a latency reversing activity (LRA) of HIV [47, 67, 68]. At 0.2 μM or 0.4 μM of JQ1 treatment, 2D10-FUS KD cells exhibited higher expression levels of their integrated d2GFP reporter compared to control 2D10 cells, indicating that upon FUS KD sensitizes, the activating activity of JQ1 on latent HIV is synthesized (twofold for 0.2 μM; Fig. 7c). To further confirm our results, early multiply spliced *Rev* and late un-spliced Gag mRNA levels were quantified by RT-qPCR in control or FUS KD 2D10 cells treated with 0.2 μM JQ1. We demonstrated that while 2D10 cells that were not treated with JQ1 displayed undetectable levels of HIV mRNA, the combination of FUS KD and treatment with JQ1 led to an increase in HIV *Gag and Rev* mRNA levels, relative to control cells that expressed FUS (Fig. 7d; twofold). We conclude that FUS modulates HIV latency and potentially promotes this state, while its KD enhances the reactivation of latent HIV following treatment with JQ1.

### FUS and AFF4 co-localize within nuclear condensates

**Fig. 8:**
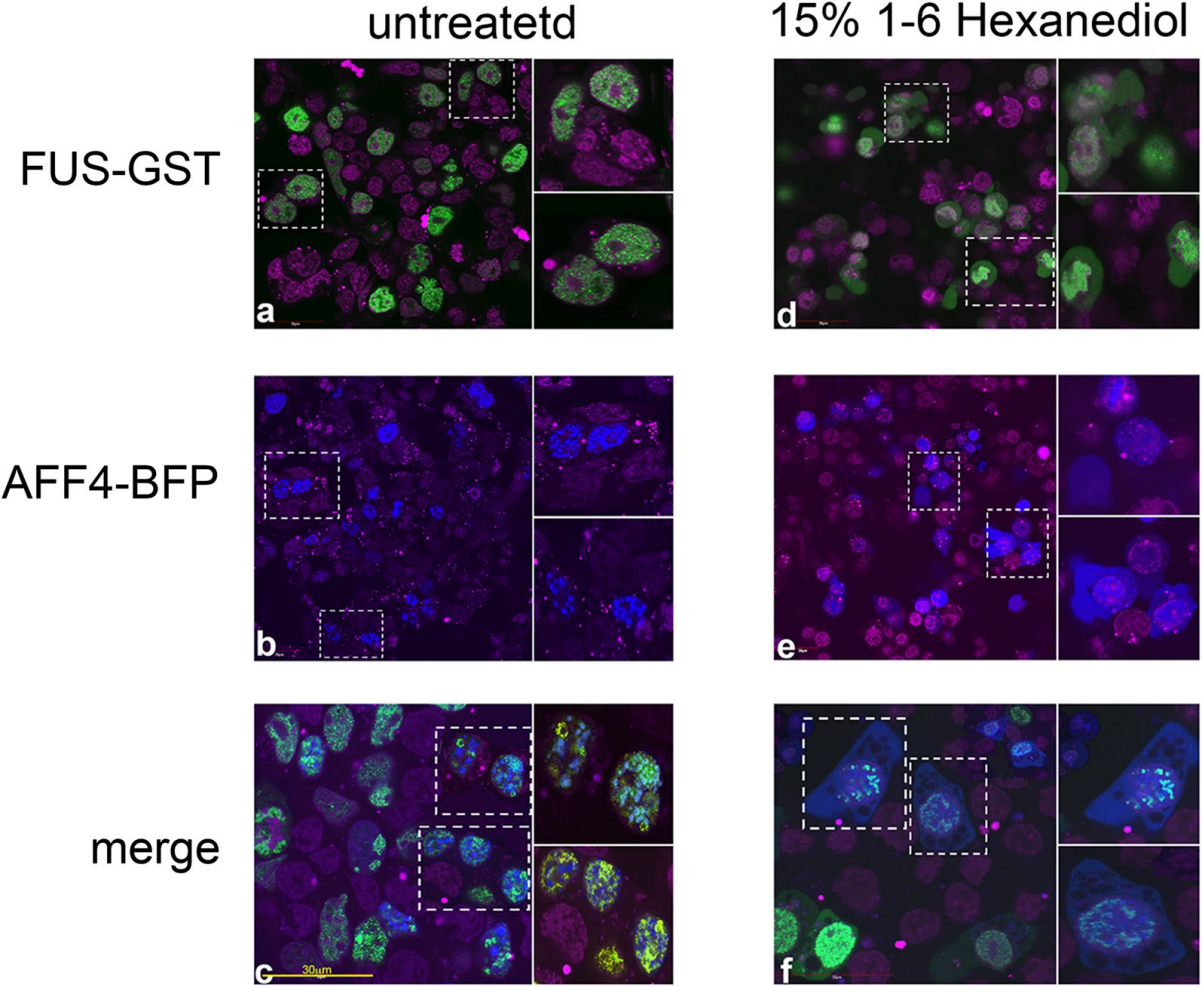
Co-localization of AFF4 and FUS within nuclear condensates. Live-imaging analysis of cells expressing either FUS-GFP (**a**), AFF4-BFP (**b**) or cells co-expressing both proteins (**c**). Cells were also treated with 1-6-Hexanediol to disrupt nuclear liquid-like condensates (**d–f**). A low resolution image is shown for each condition, where labeled cells are also displayed in a higher magnification. Cells were examined under an Olympus FV1000 confocal microscope. Nuclei were stained with DRAQ5 dye (magenta; 1:1000 dilution)

FUS is an RNA/DNA binding protein, which is primarily localized in cells to the nucleus and forms ribonucleoprotein, liquid-like nuclear droplets that phase separate from their environment. The formation of these liquid membrane-less condensates is mediated through the N-terminal low complexity (LC) region of FUS, which exhibits a disordered structure and contains multiple repeats of a S/GYS/G motifs [50]. Upon stress, FUS rapidly shuttles between these liquid compartments in the nucleus and the cytoplasm. With time, or as a result of specific mutations, FUS liquid droplets convert to an aggregated state, which is reminiscent of the pathological state seen in ALS patients [50–52, 62]. As FUS associates with AFF4 in cells, we employed live-imaging analysis to visualize their cell distribution and to determine if the proteins co-localize (Fig. 8). Cells were expressed with AFF4-BFP, or FUS-GST fusion proteins (Fig. 8a + b), or with both proteins (Fig. 8c), and those which expressed low intensity of protein fluorescence were monitored. Our imaging analysis confirmed previous reports showing that in cells, FUS exhibits a nuclear punctuated expression pattern that correspond phase separation structures (Fig. 8a) [69]. Moreover, AFF4 which also harbors low complexity motifs, was also localized to the nucleus and like FUS exhibited a granular expression pattern [70] (Fig. 8b). Upon expressing both proteins, AFF4 and FUS were co-localized into the nucleus, and displayed a condensed and punctuated expression arrangement (Fig. 8a–c). Treating cells with 15% 1,6-hexanediol, which is known to perturb weak hydrophobic interactions and disassemble membrane-less structures with liquid-like properties, partially disrupted the nuclear punctuated expression pattern of FUS and AFF4 (Fig. 8d + f). In particular, treatment also led to FUS-AFF4 protein migration to the cytoplasm, where proteins lost their punctuated expression pattern (Fig. 8d + f). We conclude that FUS co-localized with AFF4 within the nucleus and exhibits granular condensate expression patterns, similar to phase separation organelles [71].

### FUS modulates SEC occupancy genome-wide

**Fig. 9:**
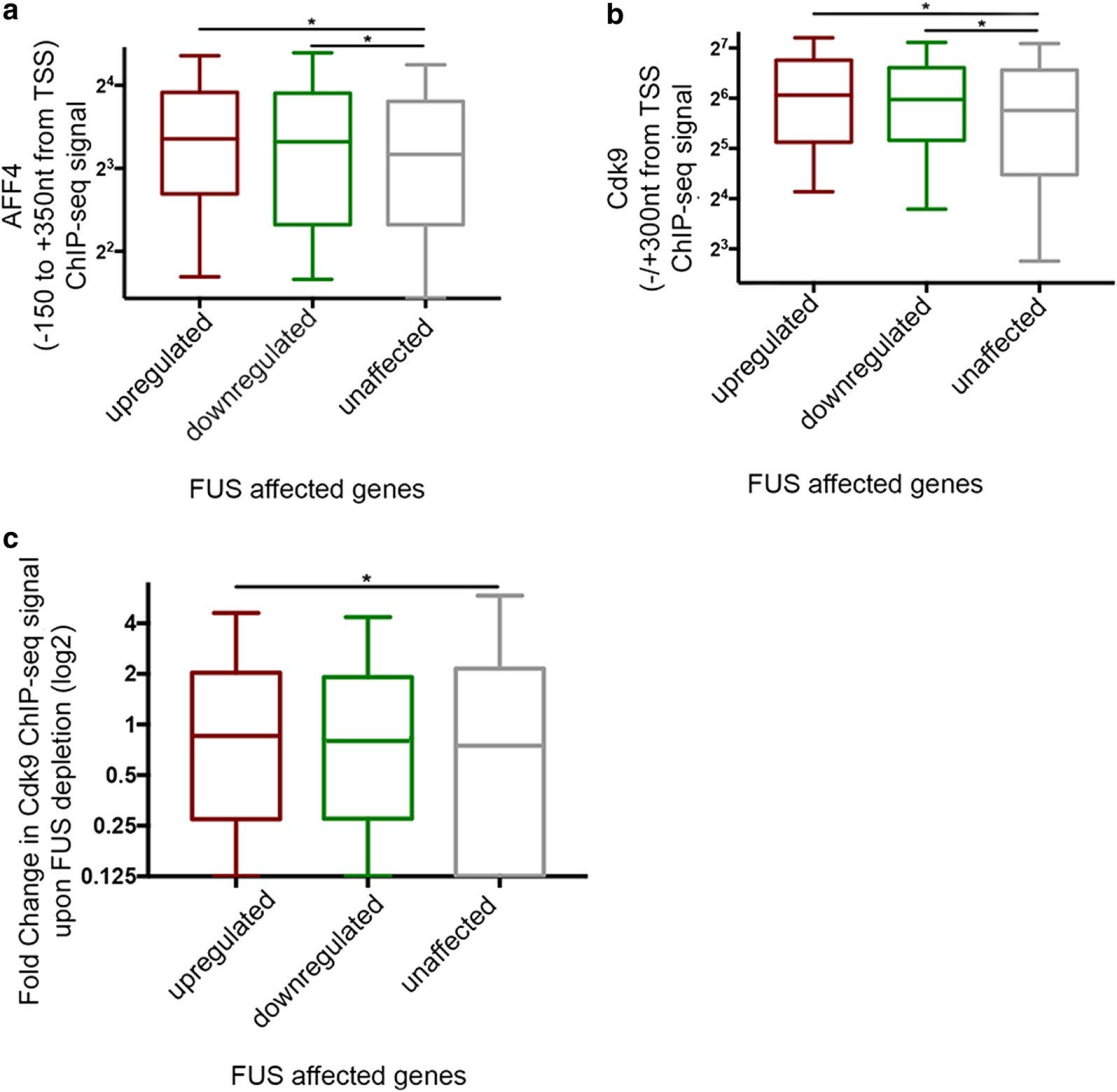
Occupancy levels of AFF4 are elevated at genes that are upregulated upon FUS depletion. **a + b** AFF4 and Cdk9 ChIP-seq occupancy dataset from untreated cells focusing on genes that are affected due to loss of FUS expression (see also Additional file 1: Figure S3 for RNA seq). For each of the indicated windows, reads of Cdk9 or AFF4 were determined. *p < 0.0001 from Kruskal–Wallis test. **c** Fold of change in Cdk9 ChIP-seq reads within genes that were affected upon FUS depletion

We next tested if the silencing effects of FUS on the occupancy of SEC and P-TEFb that were shown for the HIV promoter, were also evident on a genome wide scale. ChIP-seq analysis confirmed that like in the case of HIV, FUS is enriched around TSSs at coding genes (40) (Additional file 1: Figure S3A). This analysis called for 4141 FUS peaks, of them > 55% are located around TSSs. Interestingly, FUS is also recruited to exons (12.1%), introns (14.51%), termination sites (3.4%) and intergenic regions (14.41%) (Additional file 1: Figure S3B). We next analyzed publicly published RNA-seq datasets and defined 4194 genes that are affected upon depletion of FUS expression [29, 54]. Among these genes, 2236 were upregulated similarly to the tendency detected for HIV, while 1958 were down-regulated. 11,484 genes were found to be unaffected following FUS depletion of expression (Additional file 1: Figure S3C). We focused our analysis on FUS affected loci (in FUS KO cells), and combined these datasets with the ChIP-seq analysis describing the occupancy of SEC and P-TEFb (AFF4 and Cdk9; [29, 54]). We found that both up and down-regulated genes (upon FUS depletion) exhibited higher occupancy levels of Cdk9 and AFF4 on their promoters, relative to unaffected gene promoters (Fig. 9). Of the FUS affected genes, the upregulated genes exhibited slightly but statistically significant higher occupancy levels of both AFF4 and Cdk9 upon FUS depletion, relative to downregulated and unaffected genes (Fig. 9a–c).

As previous reports suggested that FUS associates with RNAPII-CTD and P-TEFb, we tested the effects of FUS KO on the assembly of the elongation complex—P-TEFb/SEC, RNAPII in J-LTR-Luc-FUS KO (Additional file 1: Figure S4), and compared it to that of J-LTR-Luc wild-type cells. By employing IP experiments with either Ser2 or Ser5 antibodies that target the different phosphorylated forms of the CTD of RNAPII, we demonstrated that as expected FUS associated with RNAPII, Cdk9 and AFF4/SEC in J-LTR-Luc cells. However, depletion of FUS had no effects on the association of RNAPII with P-TEFb and AFF4/SEC FUS KO cells, implying that the protein complex is assembled regardless of FUS recruitment to TSSs (Additional file 1: Figure S4). Furthermore, our results show that FUS associates both with Ser2 of RNAPII [54], and also with Ser5 of RNAPII, implying that FUS associates with the transcription machinery when the CTD is phosphorylated both on Ser5 and Ser2 (Additional file 1: Figure S4). Finally, gene ontology (GO) analysis of genes that were affected by FUS KD found that genes that were upregulated are involved in important cellular processes including cellular translational elongation, metabolism, mRNA splicing, RNA processing as well regulatory pathways of RNA. Interestingly, within the downregulated genes include negative regulation of transcription from RNAPII, demonstrating the linkage between FUS and the transcription machinery (Additional file 1: Figure S5).

## Discussion

Pivotal work on the mechanisms that control HIV gene transcription elongation has paved the way to our understating of how metazoan global gene transcription is executed [1, 11, 12]. Based on this work, the role of SEC and P-TEFb in promoting RNAPII pause-release and elongation of transcription has been well documented [5, 8, 16]. In the unique case of HIV, the viral Tat protein acts as a master regulator of transcription elongation and recruits the cellular elongation machinery to the viral promoter to enhance pause-release of RNAPII and transcription elongation [11, 12, 38]. Elucidating the role of SEC/P-TEFb in controlling HIV gene transcription is also of a high clinical significance, as transcription repression of the integrated provirus, mainly in resting CD4+ infected cells, results in the establishment and maintenance of the latent viral reservoir that is highly stable and refractory to anti-retroviral therapy. However, despite substantial progress in understanding the molecular mechanism that control HIV transcription and viral latency, a complete eradication of the cell infected reservoir is still out of reach and the role of SEC in modulating this state is yet to be defined.

In this work we identify FUS as a regulator of HIV transcription. FUS associates with AFF4 of SEC in cells and silences viral gene transcription (Figs. 1, 2, 3). We could not demonstrate direct interactions between FUS and AFF4 proteins, and hypothesize that FUS resides in the elongation complex together with RNAPII, P-TEFb and SEC. Upon ectopic expression, FUS inhibits Tat-independent HIV gene transcription. FUS also silences Tat-mediated HIV gene transcription, but to a lesser extent, implying that Tat is a more potent activator that efficiently recruits that transcription machinery. Nevertheless, the overall fold of activation of transcription by Tat has not changed upon expression of FUS due its observed decrease in basal Tat-independent transcription in the presence of FUS. Reversed effects on HIV gene transcription are also shown upon FUS depletion, which leads to enhancement of viral gene transcription (Fig. 4). However, activation of viral gene transcription following FUS depletion is detected only in the absence of Tat, while upon Tat expression HIV transcription already reaches optimal levels despite of FUS knockout (Fig. 4). These results confirm the efficiency of Tat in enhancing HIV transcription elongation. They are also consistent with our previous report demonstrating that SEC promotes Tat-independent transcription, while in the presence of Tat, SEC/P-TEFb are also recruited by the viral trans-activator to the promoter [72]. The mechanisms by which FUS is recruited to gene promoters and mediates its effects are still largely unclear. Previous reports indicate that FUS associates with the CTD of RNAPII in an RNA-dependent manner [54]. Our experiments that demonstrate that a FUS mutant that cannot bind RNA (FUS-SGG4) has no inhibitory effects on HIV transcription (Fig. 3) coincide with early reports stating that the RRM/RGG of FUS is required for FUS role in gene transcription and splicing [73]. Moreover, we show that association of the FUS-SGG4 mutant with TAR RNA is reduced relative to the wild type FUS (Additional file 1: Figure S2). Supporting this role of the FUS RNA motif, in a delta TAR HIV promoter, FUS occupancy on the viral promoter is also decreased (Fig. 6). Accordingly, our results conclude that FUS-mediated silencing is potentially promoted through inhibition of SEC/P-TEFb recruitment to the viral promoter and is dependent on RNA.

An additional possible mechanism for FUS effects would argue that the observed co-localization distribution of FUS and AFF4 within nuclear condensates, phase separated structures, might sequester the transcription machinery from gene promoters and subsequently silence transcription (Fig. 8) [50, 52, 74–76]. Previous reports show that such a mechanism exists for heterochromatin protein 1α and leads to chromatin compaction and transcription repression [77, 78]. Nuclear condensation of the polycomb repressive complex (PRC1)—CBX2 represses transcription, as it undergoes phase separation to form nuclear structures that exhibit liquid-like properties and [79]. Our data imply that AFF4 and FUS exhibit granular expression pattern similar to phase separation structures. Moreover, both protein co-localize in the nucleus. AFF4-FUS punctuated structures potentially possess liquid phase separation properties, as treating cells with hexanediol, which dissolves the hydrophobic interactions within phase separation organelles, disrupted the granular expression pattern of FUS, and AFF4, and led to protein migration into the cytoplasm (Fig. 8). As FUS is known to accumulate within phase separation structures, our working model implies that it limits the occupancy of AFF4 and subsequently SEC on gene promoters, leading to transcription silencing. The RNA binding activity of FUS is required for this effect of FUS, and current experiments investigate this hypothesis also in vitro.

Effects of FUS on HIV gene transcription are also displayed genome wide. Like in the case of HIV, FUS is globally positioned on TSSs of gene promoters. Interestingly, FUS also occupies exons and intergenic loci (Additional file 1: Figure S3), and current studies aim to analyze the role of FUS in modulating transcription of non-coding genes. Moreover, FUS globally affects SEC and P-TEFb occupancy, and in genes that are affected due to FUS knockdown, this occupancy is elevated relative to genes that are not-affected due to FUS depletion (Fig. 9; Additional file 1: Figure S3). Nevertheless, FUS knockout does not affect the assembly of the transcription machinery, and we show that it can associate with the CTD of RNAPII both at its Ser5 or Ser2 forms, implying a role of FUS both at initiation or elongation of transcription (Additional file 1: Figure S4).

Finally, the eradication of the latent HIV reservoir remains a main obstacle towards a complete cure for infection. One current approach to successfully eradicate the latent reservoir is to reactivate HIV gene expression in latently infected cells, and eliminate the active virus by HAART. To efficiently accomplish this “Shock and Kill” approach, the mechanisms that regulate HIV latency need to be fully understood, and host factors that modulate viral transcription have to be identified. Based on such studies, new drugs will be developed and will be combined with other already established regimens to optimally activate HIV gene expression. Our research shows that FUS regulates HIV latency by delaying the establishment of HIV latency state (Fig. 7). Importantly, we also demonstrate that FUS depletion enhances the reactivation of latent infected cells that is exhibited following treatment of cells with JQ1 (Fig. 7). We conclude that in our search for optimal protocols that reactivate the virus from its latent state with minimal global cell activation, targeting FUS expression should be considered as a potential therapeutic strategy that can be included with current regimens for battling HIV latency [38, 80].

## Conclusions

In this work, we identified FUS as a partner of the AFF4 subunit of SEC. We show that FUS assembles into the transcription machinery and regulates viral transcription from the viral promoter. FUS silences viral gene expression and modulates HIV latency by disrupting the recruitment of AFF4/P-TEFb to the viral promoter. Expression patterns of FUS and AFF4 within nuclear condensate assemblies within cells, may provide an additional mechanism for the role of FUS is mediating gene silencing through sequestering SEC from the HIV promoter and repressing gene transcription. These effects of FUS find their way in modulation of HIV latency, as in T cell latency models, we show that knockout of FUS delays the establishment of viral latency and enhances HIV gene activation upon treatment with JQ1. As we aim to eradicate the virally infected reservoir, understanding the molecular mechanisms that establish and maintain latency is of high clinical relevance. We propose that FUS can be considered a key factor in this process and a target for intervention, as we try to develop new strategies to eliminate HIV infection.

## Materials and methods

### Cells

Human Embryonic Kidney HEK293T (ATCC; CRL11268) that used for MS analysis were maintained in DMEM complete medium (GIBCO). Jurkat T lymphocytes cell line were maintained in RMPI medium (GIBCO), supplemented with 10% fetal bovine serum, 2 mg/ml l-glutamine, penicillin–streptomycin, and non-essential amino acids (Sigma, M7145). Cells were cultured at 37 °C with 5% CO2. J-LTR-Luc cells were generated by transducing Jurkat cells with VSV-G psuedotyped lentivirus that encodes LTR-Luciferase (Luc) reporter gene. To generate-LTR-Tat-Luc cells, J-LTR-Luc cells were further transduced with a lentivirus encoding HA-Tat lentivirus and the blue fluorescent protein (BFP) under the control of the HIV LTR promoter. Clonal population was obtained after sorting single cells expressing BFP by FACS. To ectopically express FUS, the indicated cells were transduced with a lentivirus encoding the for Flag-FUS under the CMV promoter, followed by puromycin selection. 2D10 are Jurkat T cells that express HIV-LTR-2dGFP and represent latent cell model as described in the text [66].

### Plasmids

FUS expression plasmid was described previously [54]. FUS cDNA was sub-cloned as a Flag-tagged protein in pCDNA3, or into a lentiviral vector. For analysis of HIV gene transcription, lentiviral vector expressing the Luciferase Protein under the LTR promoter (HIV-LTR-Luc) reporter, regulated by HIV-LTR-was used. HA-AFF4 expression plasmid was a generous gift from Dr. Qiang Zhou laboratory—University of California, Berkeley. HA-AFF4 was sub-cloned into a lentiviral vector harboring N-terminal HA-tag. Constructs for truncated HA-AFF4 proteins were generated by PCR amplification of the desired length of AFF4 cDNA, using an N-terminal primer and primers located at the C-terminus region. CRISPR/Cas9 expression plasmids were obtained from Addgene (#49535).

### Production of VSV-G pseudotyped lentiviruses

Single round viral particles were produced by calcium phosphate-mediated co-transfection of the lentivector expressing the pLTR-Luciferase, and plasmids coding for HIV structural and regulatory genes (gag/pol, Rev, Tat) and VSV-G envelope into packaging cell line Human Embryo Kidney HEK 293T. Viral particles were harvested from culture supernatant 48 h post transfection, spun at 2000 rpm for 10 min to remove cell debris, and filtered through 0.45 μm filter (Amicon). Lentiviral particles were concentrated by ultra-centrifugation for 2.5 h at 25,000 rpm, (Beckman OptimaL 90 K ultracentrifuge, SW-28 rotor) and the pellet was re-suspended in PBS. Titer of lentiviruses encoding for the Luc reporter was determined by transduction of Jurkat cells with serial dilutions of the virus stock followed by Luc reporter assays.

### Lentiviral transduction and luciferase reporter assays

Cells were transduced with VSV-G pseudotyped lentivirus expressing the HIV-LTR luciferase transgene. Similarly, lentivirus that express FUS or Cas9/sgRNA were used for transduction. 48 h post transduction, cells were harvested and their luciferase activity was measured according to the manufacture manual (Promega). Luciferase readings were normalized to protein expression or to *Renila* as an internal control, and are presented relative to the readings obtained in parental Jurkat cells—set to 1. Results are presented as the mean value of triplicate wells; error bars show ± SEM.

### CRISPR-mediated gene silencing

J-LTR-Luc cells were transduced with lentivirus encoding Cas9 and each of two different sgRNAs that target FUS (Addgene #49535). A mixture of the two guides were transduced. As a control, cells were also transduced with the same Cas9 encoding virus where a scranbled sgRNA was cloned. Following lentiviral transduction, cells were cultured with media supplemented with 1 μg/ml of puromycin to eliminate non-transduced cells. Single puromycin resistant clones were obtained by serial dilution in a 96 well and further expanded. Clones were genotyped to confirm gene editing, and depletion of FUS expression was further confirmed by western blotting analysis with specific anti-FUS antibody (4H11; Santa Cruz Biotechnology #47711). Two different clones of cells that negatively express FUS (J-LTR-Luc FUS KO #1 and #11) were functionally analyzed. Western blot analysis with anti-Tubulin antibody confirmed equal loading of protein.

### Purification of HA-AFF4 associating proteins from cells

HEK293T cells were grown on 10 cm culture dishes and were transfected with 10 μg of HA-AFF4 (Full length, 300 residues) using Lipofectamin2000. 2 μg of a plasmid expressing a GFP reporter gene was also co-transfected for measuring transfection efficiency. 48 h post transfection, 2 × 10^9^ HEK 293T cells expressing HA-tagged AFF4 proteins were harvested and lysed with optimized IP buffer (500 μl; 0.15% Triton X-100; 20 mM Tris–HCl-pH 7.6; 200 mM NaCl; 0.72 mM EDTA and 10% Glycerol; supplemented with protein inhibitor cocktail—added fresh before use and phosphatase inhibitors: 6 mM of NaF and 2 mM of Na_2_VO_4_). Cell lysates were then incubated for 1 h on ice before centrifuged 14,000 rpm for 5 min at 4 °C. Supernatant was then collected and cleared with protein A Sepharose beads before incubation with 5 μg of anti-HA antibody (Abcam) overnight at 4 °C with gentle rocking. The next day, lysates were incubated with 50 μl of pre-blocked protein A beads were added to IP samples and incubated on a rocker for additional 2 h at 4 °C. Beads were washed × 4 times with the IP buffer supplemented with 0.1% Triton X-100 and centrifuged for 3 min at 3000 rpm at 4 °C. 5% of the eluate was resolved on SDS-PAGE and proteins were visualized by silver staining using a Silverquest kit (Invitrogen). The remaining eluate was resolved on SDS-PAGE, then stained with Coomassie-R250. Individual Coomassie-R250 stained bands were excised and proteins in gels digested with sequencing grade trypsin according to the manufacturer’s protocol (Promega). Excised gel pieces were first washed with 50% acetonitrile in 50 mM ammonium bicarbonate, and then dehydrated with acetonitrile. Proteins were then subjected to overnight proteolytic digestion (after reduction in 20 mM DTT at room temperature for 2 h and alkylation with 50 mM iodoacetamide in 50 mM ammonium, bicarbonate for 1 h in the dark), followed by extraction from the gel with 5% formic acid in 50% acetonitrile and then re-suspended in 0.1% formic acid after being dried under vacuum. Samples were then analyzed by tandem Mass Spectrometry at BGU Nanotechnology facility.

LC/MS analysis of the protein digests was performed using an Eksigent nano‐HPLC (model nanoLC-2D, Netherlands) connected to the LTQ Orbitrap XL ETD (Thermo Fisher Scientific, Germany and USA). Reverse‐phase chromatography of peptides was performed using homemade C‐18 column (15 cm long, 75 μm ID) packed with Jupiter C18, 300 Å, 5 μm beads (Phenomenex). Peptides were separated by a 70‐min linear gradient, starting with 100% buffer A (5% acetonitrile, 0.1% formic acid) and ending with 80% buffer B (80% acetonitrile, 0.1% formic acid), at a flow rate of 300 nl/min. A full scan, acquired at 60,000 resolution, was followed by CID MS/MS analysis performed for the five most abundant peaks, in the data‐dependent mode. Fragmentation (with minimum signal trigger threshold set at 500) and detection of fragments were carried out in the linear ion trap. Maximum ion fill time settings were 500 ms for the high‐resolution full scan in the Orbitrap analyzer and 200 ms for MS/MS analysis in the ion trap. The AGC settings were 5 × 10^5^ and 1 × 10^4^ (MS/MS) for Orbitrap and linear ion trap analyzers, respectively. Proteins were identified and validated using the SEQUEST and Mascot search engine operated under the Proteome Discoverer 1.4 software (Thermo Fisher Scientific) Mass tolerance for precursors and fragmentations was set to 10 ppm and 0.8 Da, respectively. Only proteins containing at least two peptides of high confidence (Xcore N 2 or 2.5 for doubly or triply charged species, respectively) were chosen.

### Immunoprecipitation in cells

For mapping the regions in AFF4 that interact with FUS, HEK293T cells were transfected with plasmids encoding Flag-FUS and the each of the indicated HA-AFF4 C-terminal truncated proteins. Cells were then harvested and lysed with 500 μl lysis buffer (0.15% Triton X-100; 20 mM Tris–HCl pH 7.6; 200 mM NaCl; 0.72 mM EDTA; 10% Glycerol; 1 mM DTT, supplemented with protease inhibitors cocktail (Sigma; 1:200 dilution). Lysates were pre-cleared with Protein A-Sepharose beads (Invitrogen) and then incubated on ice for 1 h and centrifuged at 14,000 rpm for 10 min at 4 °C. Cleared supernatants were then incubated overnight with gentle rocking with 1 μg of anti-HA antibody (Abcam #9110). 50 μl was taken of the lysis before the addition of anti-HA antibody for input analysis. Expression levels of Flag-FUS were monitored with the anti-Flag antibody (M2-Sigma; A2220).

### Cell-based latency assays

J-LTR-Luc cells or cells where FUS expression was depleted (J-LTR-Luc FUS KO) were infected at day 0 with pseudotyped HIV-GFP virus (pHR-GFP HIV). Cells were then sorted for their GFP expression and further cultured for the indicated time days post infection to gradually enter latency. GFP expression was monitored in control or FUS KO cells at the indicated time points. To ensure that cells in the pooled population harbor latent provirus. Cells at day 60 post infection were analyzed by FACS, and the GFP(−) expressing cells were sorted and treated with JQ1. 2D10 T cells were transfected with either control scramble siRNA or siRNA specific against FUS (synthesized by IDT) by Neon electroporation system (Thermo). Following transfection of cells, growth media was changed and let grow for additional 72 h post transfection. Cells were stimulated with JQ1 at the indicted concentration for 24 h and GFP expression was monitored by FACS. Specific HIV RNA was also extracted from control and FUS KD 2D10 cells and was monitored by quantitative PCR (qPCR) using specific primers positioned on *Gag* (non-spliced HIV mRNA) and *Rev* (multiply spliced HIV mRNA).

Rev primers were used as described [81].

Forward GAAGAAGAAGGTGGAGAGAGAGAC.

Reverse TGTAGCAAGCTCGATGTCAGCAGT.

Gag primers were also previously described [82].

### Chromatin immunoprecipitation analysis (ChIP) qPCR analysis

J-LTR-Luc and J-LTR-Luc FUS KO cells were cross-linked with 1% formaldehyde for 10 min, and after washing with PBS, cross-linking was stopped by adding glycine (0.125 M; 5 min). Cells were then lysed for 10 min on ice in 500 μl lysis buffer (50 mM HEPES pH-7.5, 140 mM NaCl, 1% Triton X-100, 1 mM EDTA, 0,1% SDS and 1% protease inhibitor cocktail;) and the nuclear pellets were collected. DNA was fragmented by sonication with the following settings: amplitude 40%, for 10 cycles 20 s on/40 s off)Sonics Vibra Cell(. Samples were centrifuged (15 min, 14,000 rpm, 4 °C) and the soluble chromatin fraction (50 μg) was collected and immune-precipitated overnight with either anti-FUS IgG (5 μg), anti-HA IgG or anti-Cdk9 IgG. Precipitated DNA fragments were extracted with phenol–chloroform and quantified by qPCR with the primers specific to the LTR promoter (Forward:_5′—AGGTTTGACAGCCGCCTA-3; Reverse: AGAGACCCAGTACAGGCAAAA). All signals were normalized relative to the input DNA. ChIP assays were also performed with normal rabbit or mouse IgG as negative controls. Methods for the whole-genome ChiP-Seq and RNA-seq analysis are described in the Additional files.

### Live-cell imaging

HEK293T cells were grown in 1 cm gelatin coated micro-slides and transfected 0.1 μg expression plasmids. Prior to imaging, cells were sorted for low GFP/BFP expression to eliminate over expression effects on protein localization. 48 h post transfection hexanediol was added to the medium to reach a final concentration of 15% for 2 min and thereafter cells were washed and media was added. Live imaging was taken with FV1000 confocal microscope (Olympus) at magnification of X60. Nuclease staining was performed using DraQ5 dye (Thermo Fisher), diluted 1/1000 in PBS.

## Additional files

**Additional file 1.** Supporting results.

**Additional file 2.** Summary of MS analysis - HA-HFF4-full-length.

**Additional file 3.** Sumamry of MS analysis - HA-HFF4-(1-300).

## Data Availability

All data generated or analyzed during this study are included in this published article [and its additional file].

## Abbreviations

HIV: human immunodeficiency virus
SEC: super elongation complex
AFF4: AF4/FMR2 family member 4
P-TEFb: positive transcription elongation factor b
RNAPII: RNA polymerase II
ELL2: elongation factor for RNA polymerase II 2
FUS: fused in sarcoma
DSIF: DRB sensitivity inducing factor
NELF: negative elongation factors
DRB: 5,6-dichlorobenzimidazone-1-β-D-ribofuranoside
TSSs: transcription starting site
CTD: C-terminal domain
PAFc: human polymerase-associated factor complex
KD: knockdown
KO: knockout
ART: antiretroviral therapy
EWS: Ewing’s sarcoma
TAF15: TATA-binding protein-associated factor
ALS: amyotrophic lateral sclerosis
MS: mass-spectrometry
