# Supplementary material for: Fused in sarcoma silences HIV gene transcription and maintains viral latency through suppressing AFF4 gene activation
Source: Retrovirology. 2019 Jun 25;16:16. doi: 10.1186/s12977-019-0478-x (PMC6593535; doi:10.1186/s12977-019-0478-x)
Supplement: Supplementary file 1 — Additional file 1. Supporting results. [file 12977_2019_478_MOESM1_ESM.docx]

**Additional files**

| **Protein** | **Unique Peptides** | **Coverage (A2/A4)** | **XCorr** |
| --- | --- | --- | --- |
| AF4/FMR2 family member 4 OS=Homo sapiens GN=AFF4 PE=1 SV=1 - [AFF4_HUMAN] | IPSQPLDASASGDVSCVDEILK | High | 4.86 |
|  | LSSEHYSSQSHGNSMTELKPSSK | High | 3.86 |
|  | LSSEHYSSQSHGNSMTELKPSSK | High | 3.67 |
|  | LKIPSQPLDASASGDVSCVDEILK | High | 3.67 |
|  | APSSSSNCPPSAPTLDSSKPR | High | 3.63 |
|  | VPFSSGQHSTQSFPPSLMSK | High | 3.45 |
|  | EKAPSSSSNCPPSAPTLDSSKPR | Medium | 3.11 |
|  | SNSMLQKPTAYVRPMDGQESMEPK | Low | 2.88 |
|  | SNSMLQKPTAYVRPMDGQESMEPK | Low | 2.82 |
|  | LVAIPKPTVPPSADEK | High | 2.77 |
|  | DPDANWDSPSR | High | 2.70 |
|  | SNPNFFEQR | Low | 2.06 |
| Cyclin-T1 OS=Homo sapiens GN=CCNT1 PE=1 SV=1 - [CCNT1_HUMAN] | RGPSEETGGAVFDHPAK | High | 3.96 |
|  | TSENLALTGVDHSLPQDGSNAFISQK | High | 3.59 |
|  | IKVHAAADKHNSVEDSVTK | Medium | 3.34 |
|  |  |  |  |
|  |  |  |  |
| Cyclin-dependent Kinase 9 OS=Homo- Sapiens | IDSDDALNHDFFWSDPMPSDLK | High | 3.90 |
|  | HENVVNLIEICR | High | 3.62 |
|  | NPATTNQTEFER | High | 3.49 |
|  | DPYALDLIDK | High | 3.08 |
|  | VVTLWYRPPELLLGER | Medium | 3.06 |
|  | IGQGTFGEVFK | High | 2.67 |
|  | LADFGLAR | Medium | 2.58 |
|  | AANVLITR | Medium | 2.45 |
| RNA Binding Protein FUS OS=Homo Sapiens | HDSEQDNSDNNTIFVQGLGENVTIESVADYFK | High | 6,29 |
|  | APKPDGPGGGPGGSHMGGNYGDDR | High | 3,51 |
|  | AAIDWFDGKEFSGNPIK | High | 3,41 |
|  | APKPDGPGGGPGGSHMGGNYGDDR | Low | 2,86 |
|  | GEATVSFDDPPSAK | High | 2,68 |

**Figure S1: Identification of HA-AFF4-full length partners**

HA epitope-tagged AFF4-Full-length (1-1163) protein was purified with anti-HA IgG from cells. Bound proteins were separated by SDS-PAGE and visualized by silver staining. Cellular partners of HA-AFF4-full length were determined by mass spectrometry. Presented are recovered peptides from the IP with HA-HFF4-full-length that were previously confirmed as AFF4 protein partners, and were detected by the MS analysis. For each protein, number of unique peptides and their sequence are depicted, as well as the peptide percentage of protein coverage (the percent coverage calculated by dividing the number of amino acids in all found peptides by the total number of amino acids in the entire protein sequence) and the XCorr values (the number of fragment ions that are common to two different peptides with the same precursor mass and calculates the cross-correlation score for all candidate peptides queried from the database by SEQUEST searches).

**
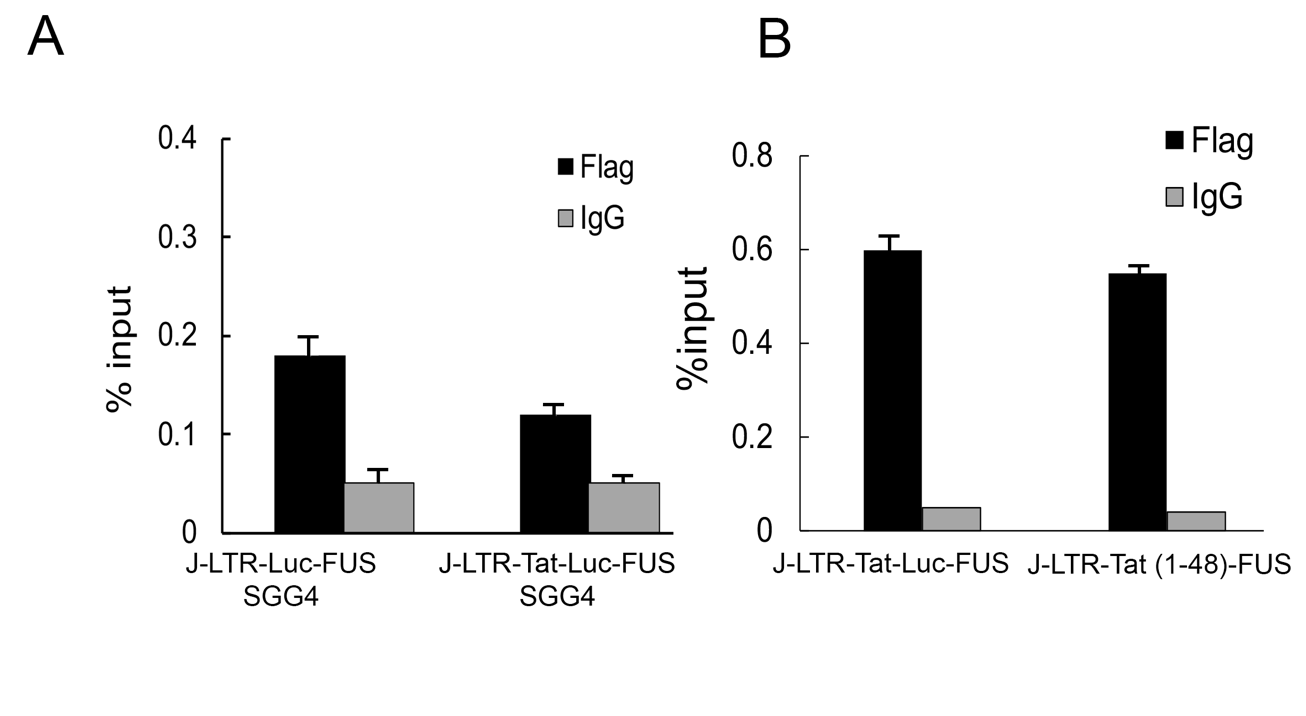
**

**Figure S2: Protein RNA interaction analysis**

1. **Association of FUS with TAR RNA is dependent on its RNA binding activity**

Jurkat **(**J)-LTR-Luc-FUS-SGG4 and J-LTR-Tat-FUS-SGG4 cells that harbor the integrated HIV-LTR-Luc reporter and also over-express Flag-FUS-SGG4 mutant that does not bind RNA were subjected to RNA immunoprecipitation (RIP) qPCR analysis. Cell lysate was immuno-precipitated with either anti-Flag-IgG (black bars), or control non-specific IgG (gray bars). RNA was extracted from IP or input (10%) samples with Trizol, followed by reverse transcription and amplification with primers that target the indicated RNA. qPCR reactions on samples were performed in triplicates and presented as percentage from input ChIP material. The error bars represent mean +/- SD of the triplicate independent qPCR reactions. Asterisks indicate levels of statistical significance calculated by two-tailed student T test (p<0.01).

1. **A Tat mutant that does not bind to TAR cannot compete with FUS on binding to TAR**

Jurkat **(**J)-LTR-Tat-Luc-FUS and J-LTR-Tat [delta RRM]-FUS, where Tat lacks its its RRM motif (Tat-1-48, lacks TAR binding). Cells were subjected to RNA immunoprecipitation (RIP) qPCR analysis. Cell lysate was immuno-precipitated with either anti-Flag-IgG (black bars), or control non-specific IgG (gray bars). RNA was extracted from IP or input (10%) samples with Trizol, followed by reverse transcription and amplification with primers that target the indicated RNA. qPCR reactions on samples were performed in triplicates and presented as percentage from input ChIP material. The error bars represent mean +/- SD of the triplicate independent qPCR reactions.


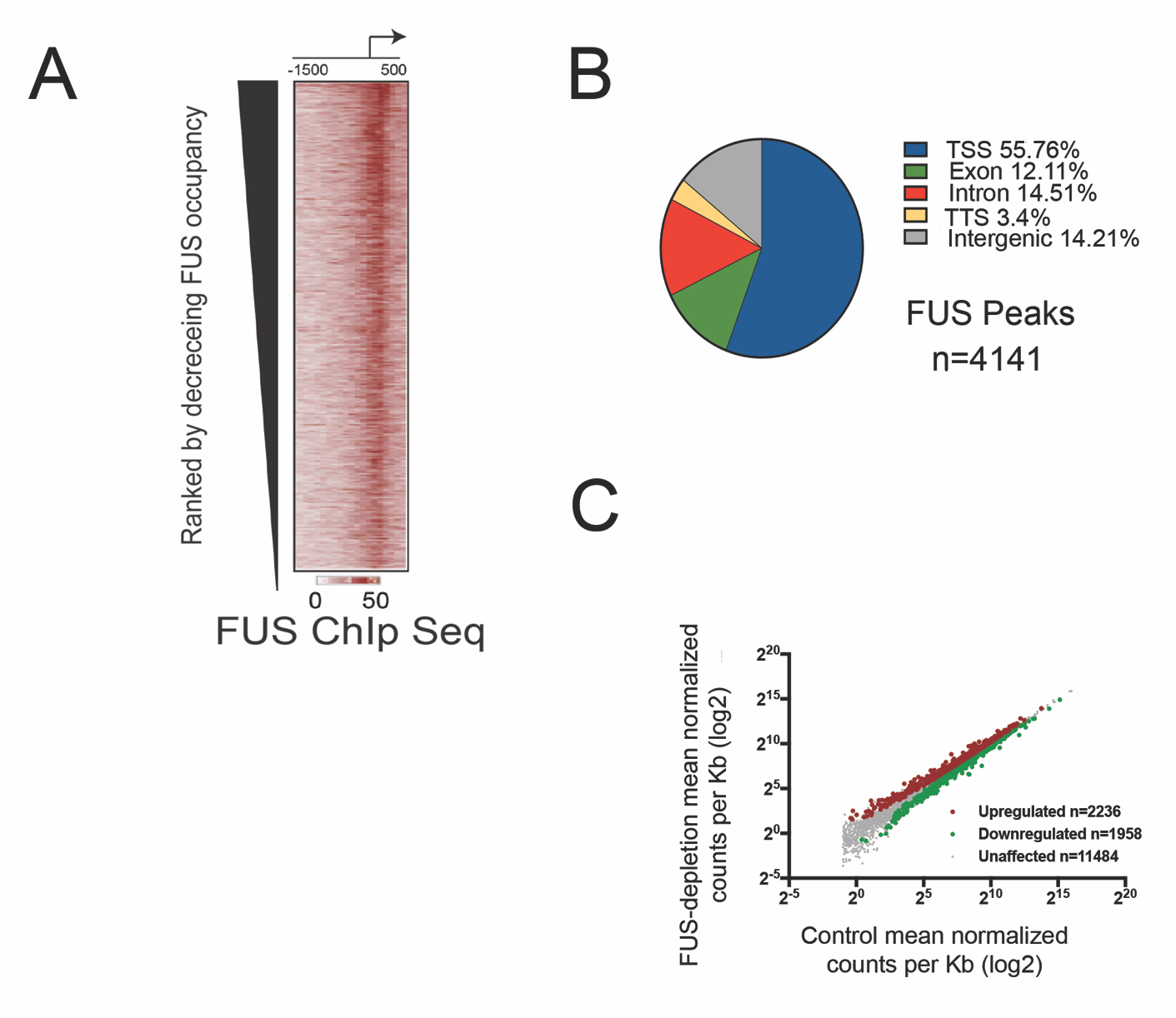


**Figure S3: FUS occupies TSS genome-wide** **and its depletion leads to change in gene expression**

1. **FUS occupies TSS genome-wide**

FUS-ChIP-Seq analysis from HEK293T cells using anti-FUS antibody, indicating the recruitment of FUS to TSSs around coding genes. ChIP-seq dataset is available at GEO (GSE34097) (39). Data is oriented around TSSs shown as arrow. Genes are ranked according to the normalized data and are ordered based on number of reads corresponding occupancy of FUS around TSS.

1. Pie chart showing the percentage of FUS occupancy along the different regions of genes. Peak calling from FUS ChIP-seq datasets performed in HEK cells to monitor the distribution of the protein in the genome (39). To position FUS along the different gene regions, Pol II ChIP-seq datasets were taken from Lou *et.al* (22) and analyzed by HOMER *de-novo* motif analysis, which reveals that FUS peaks coincide along genes around TSS, intragenic sequence, intron, exons and TTS.
2. **Defining genes that are affected by FUS depletion** - RNA-seq analysis defines three gene groups where there is a broad reduction (downregulated), activation (upregulated) in gene transcription, as well as non-affected genes. RNA-seq dataset is publicly available (22). Analyzing only mRNA genes, a FPKM >0.5 (threshold commonly used to define expressed genes) and p adjusted value < 0.05 we identified 2,236 upregulated, 1,958 downregulated genes out of 15,678 mRNA expressed genes in HEK293T cells.


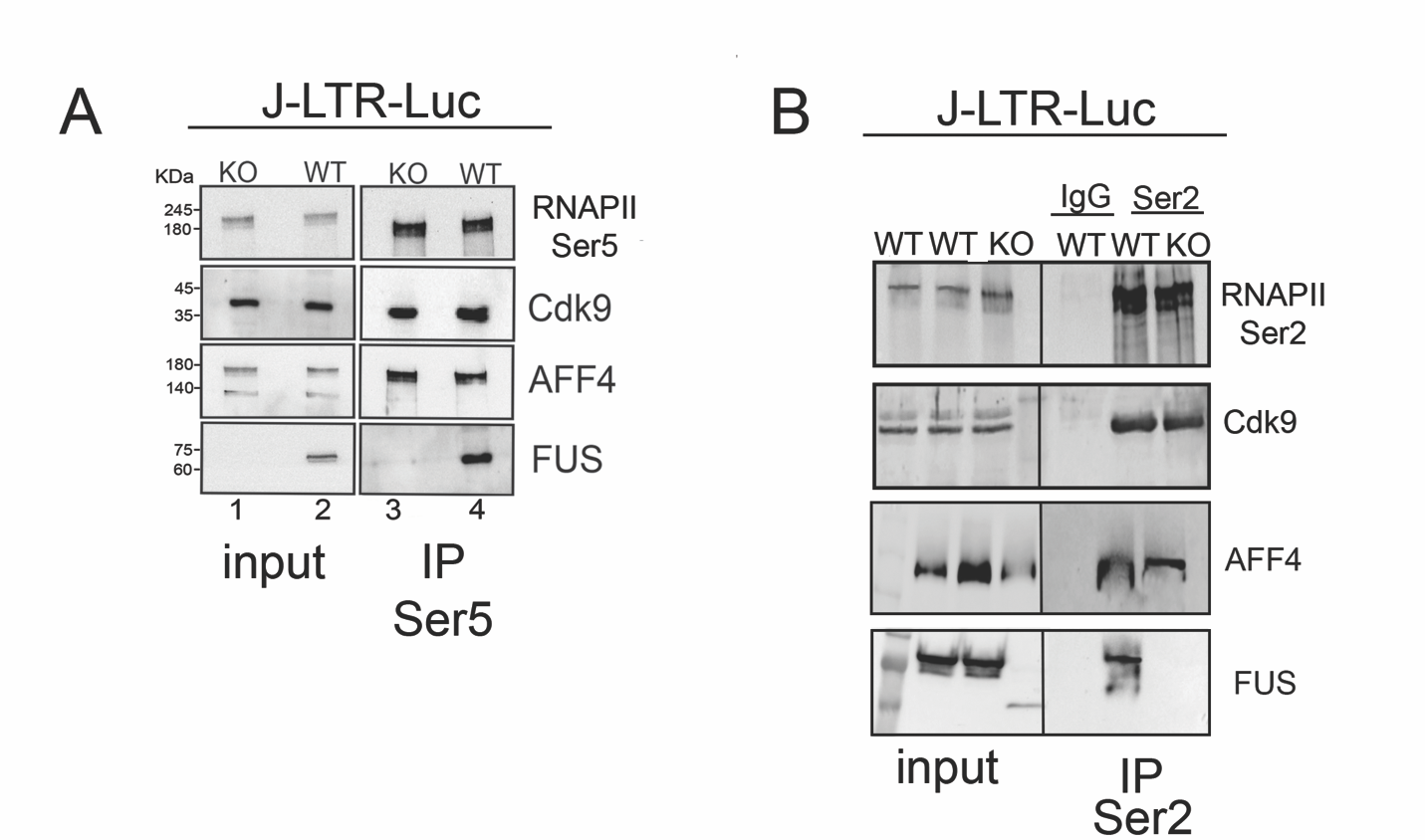


**Figure S4: Association of FUS with the elongation machinery**

Cell extract from J-LTR-Luc or J-LTR-Luc-FUS-KO was subjected to IP with either anti-RNAPII CTD Ser5 antibody (**A**; ab4H8), or anti-RNAPII-CTD-Ser2 (**B**; ab5095). IP samples were then incubated with protein A sepharose beads, and analyzed by SDS-PAGE followed by western blot with the indicated antibodies. Input represent 5% of total cell lysate.

**
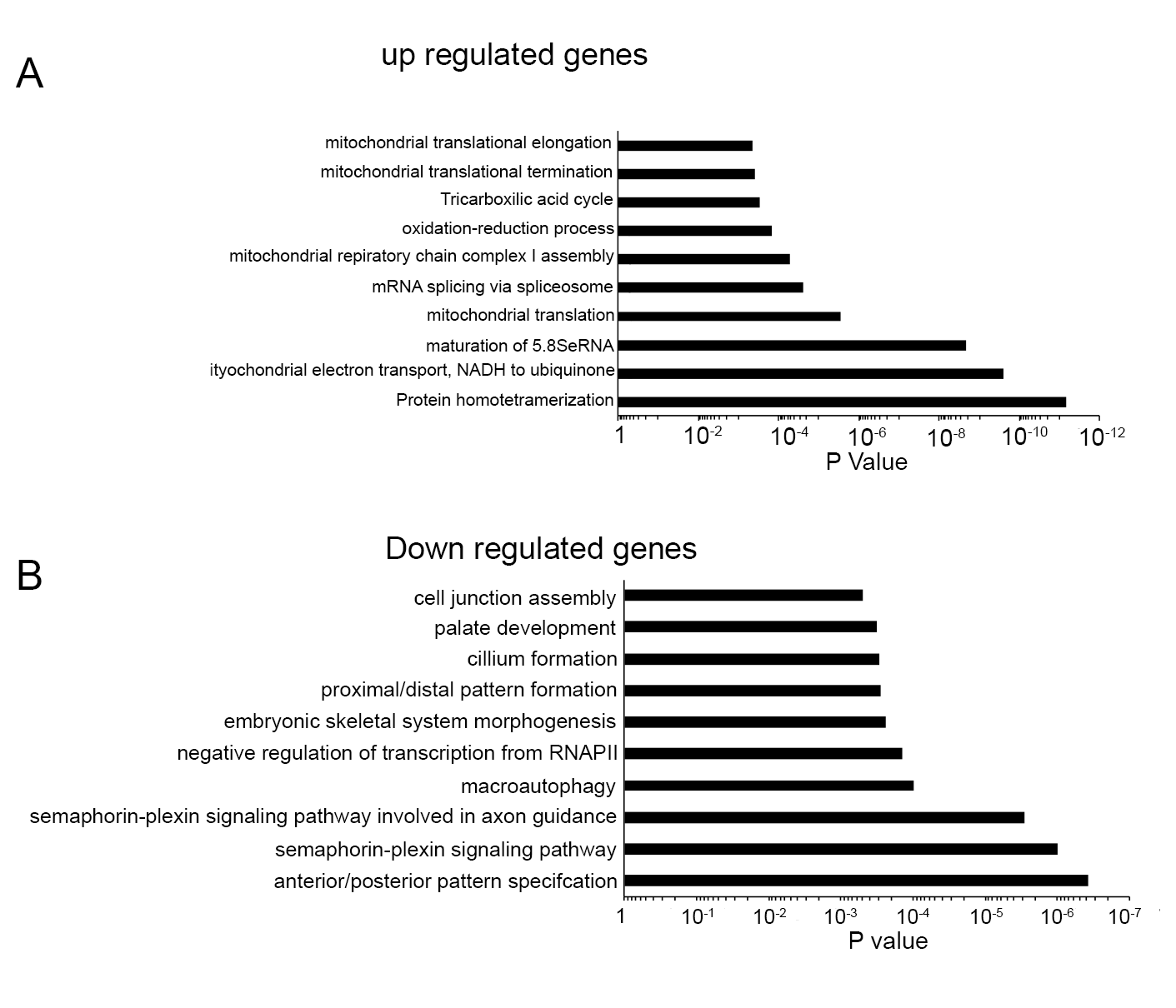
**

**Figure S5: Gene Ontology (GO) terms enriched in FUS affected genes of FUS affected genes**

Shown are representative plots of Gene Ontology analysis of genes that are either down or upregulated upon FUS depletion. Categories and p-values are presented. Gene Ontology analysis was performed using an online tool (Database for Annotation, Visualization and Integrated Discovery (DAVID) v6.7 (https://david.ncifcrf.gov/home.jsp) and standard parameters.

**Figure legend - additional files Table 1 and 2**

**Table 1+2: MS analysis of cells stably expressing AFF4**

Cells expressing either HA epitope-tagged AFF4-Full-length (1-1163 ; Table 1) or AFF3-(1-300; Table 2),were lysed and cell HA-AFF4 was purified with anti-HA IgG. Bound AFF4-associated proteins were separated by SDS-PAGE and visualized by silver staining. Cellular partners of HA-AFF4 or AFF4-300 were determined by mass spectrometry. Presented are recovered peptides from the IP with HA-HFF4-full-length or AFF4-(1-300). For each protein, number of unique peptides and their sequence are depicted, as well as the peptide percentage of protein coverage (the percent coverage calculated by dividing the number of amino acids in all found peptides by the total number of amino acids in the entire protein sequence) and the XCorr values (the number of fragment ions that are common to two different peptides with the same precursor mass and calculates the cross-correlation score for all candidate peptides queried from the database by SEQUEST searches). See additional information - Figure 1.

**Supporting Methods**

**Association of FUS with the elongation machinery RNAPII, AFF4 and P-TEFb**

For analyzing the association of FUS with the elongation transcription machinery (AFF4 and Cdk9 and RNAPII) or the effects of FUS on assembly of the elongation machinery, cells were lysed in TNEN lysis buffer (20 mM Tris-HCl (pH 7.6), 100mM NaCl, 2mM EDTA, 10% glycerol, 7 mM DTT, 1% NP-40 protease inhibitor mixture (Sigma, added fresh before use at a ratio of 1:200). Lysate was pre-cleared with Protein A-sepharose beads (Invitrogen) and then incubated overnight with 5μg of either anti RNAPII antibody (ab5408; anti-RNA polymerase II CTD repeat YSPTSPS (phospho Ser5) antibody [ab 4H8]; anti-CTD-Ser2 IgG [ ab#5095] followed by control IgG -rabbit-HRP – Jackson #12023); Cdk9 (ab6544); or FUS antibody (4H11; sc47711) at 4°C. Following, IP samples were incubated with BSA-pre-blocked protein A beads at 4°C for 2 hours with gentle rocking. Beads were then extensively washed x3 times with washing buffer (lysis buffer containing 0.05% Triton X100) and were then precipitated by centrifugation at 3000 rpm at 4°C for 5 minutes. Samples were then heated at 95ºC for 5 minutes in Laemmli sample buffer and resolved by SDS-PAGE, followed by western blot analysis using the indicated antibodies: anti-AFF4 (ab57077); anti Cdk9 (ab6544), anti RNAPII CTD Ser5 (ab-4H8) and Ser2 (ab5095) and anti FUS (4H11; sc47711).

**ChIP seq**

For ChIP-Seq, 7.5-8x10^6^ HEK293T cells were crosslinked for 10 minutes with 1% formaldehyde. For the ChIP-seq libraries, separate immunoprecipitations were performed with 12μl anti-FUS antibody. Immuno-precipitated material was purified using the Qiagen-quick PCR purification kit and ChIP-Seq libraries were prepared using the NEXTflex ChIP-seq kit (Bio-Scientific) according to the manufacturer’s instructions. ChIp-seq data were obtained from Schwartz *et.al* (39), accession number PRJNA185008.

**Publicly Available NGS Data**

Pol II and AFF4 ChIP-seq from HEK293 cells was published previously (22) and is available for downloading from GEO (GSE34097). Data was analyzed as described previously (22,39) and normalized by uniquely mapped reads. FUS and CDK9 ChIP-seq from HEK293 cells was published previously (39) and was downloaded from GEO (BioProject 185008). Data was analyzed as described previously and normalized by uniquely mapped reads.

**RNA-Seq**

RNA-seq from siNEG or siFUS treated HEK293 cells was published previously (39) and was downloaded from GEO (BioProject 185008). Data was analyzed as described previously and normalized using DESeq2 size factors (see below for more details).

Sequencing reads of RNA-seq were also obtained from Schwartz *et.al* (39). (NEG_RNAseq SRR648319 siFUS_RNAseq SRR648320. Sequencing reads were filtered (requiring a mean quality score ≥20), trimmed to 40nt, and then mapped to the hg19 reference genome using STAR 2.5.2b. Default parameters were used except that multimappers were reported randomly (outMultimapperOrder Random), spurious junctions were filtered (outFilterType BySJout), minimum overhang for non-annotated junctions was set to 8 nucleotides (alignSJoverhangMin 8), and non-canonical alignments were removed (outFilterIntronMotifs RemoveNoncanonicalUnannotated). Read counts were calculated per gene, in a strand-specific manner, based on RefSeq annotations (hg19 genome build), using featureCounts (Y. Liao, G. K. Smyth, W. Shi, featureCounts: an efficient general purpose program for assigning sequence reads to genomic features. Bioinformatics 30, 923-930 (2014)). Differentially expressed genes were identified using DESeq2 v1.18.1 (S. Anders, W. Huber, Differential expression analysis for sequence count data. Genome Biol 11, R106 (2010)) under R 3.3.1. RNA-seq size factors were determined based on DESeq2. At an adjusted p-value threshold of < 0.05, 4,194 mRNA genes (2,236 upregulated and 1,958 downregulated) were identified as differentially expressed upon FUS depletion in HEK293 cells. UCSC Genome Browser tracks displaying mean read coverage were generated from the combined replicates per condition, normalized as in the differential expression analysis.

**FUS Peak Calling**

Peak Calling was performed on the FUS ChIP-seq dataset with HOMER v4.9. HOMER was run with standard options (-style histone) and the siFUS ChIP-seq dataset was used as background (-i <input>). 4,141 peaks were called and annotated with the software package provided script 'annotatePeaks.pl'. The latest build of hg19 genome was used as reference.

## Gene Ontology (GO) Analysis

Gene Ontology analysis was performed for RNA-seq dataset of FUS affected genes using DAVIDand standard parameters (Database for Annotation, Visualization and Integrated Discovery (DAVID) v6.7 (<https://david.ncifcrf.gov/home.jsp>). Ranked p-values (log 10) represent the closeness of GO enrichment (62,63).
